# Ticks as vectors of Trypanosomatidae with medical or veterinary interest: Insights and implications from a comprehensive systematic review and meta-analysis

**DOI:** 10.1016/j.heliyon.2024.e40895

**Published:** 2024-12-05

**Authors:** Tahar Kernif, Bachir Medrouh, Naouel Eddaikra, Bruno Oury, Philippe Holzmuller, Denis Sereno

**Affiliations:** a Laboratory of Parasitic Eco-Epidemiology and Population Genetics, Pasteur Institute of Algeria, Dely-Brahim, Algiers, Algeria; b Research Centre for Agropastoralism, Djelfa, 17000, Algeria; c UMR ASTRE, CIRAD, INRAE, University of Montpellier (I-MUSE), Montpellier, France; d UMR INTERTRYP, IRD, CIRAD, University of Montpellier (I-MUSE), GoInsect: Infectiology and Entomology Research Group, Montpellier, France

**Keywords:** Trypanosomatidae, *Trypanosoma*, *Leishmania*, Ticks, Ixodidae, Argasidae, Vector borne disease

## Abstract

Since the 20th century, numerous studies have detected or isolated parasites from the Trypanosomatidae family in various tick species. However, the status of ticks as vectors for medically or veterinary significant *Trypanosoma* and *Leishmania* remains unclear. We conducted a systematic review and meta-analysis to provide new insights into the potential vector status of these pathogens, which have significant medical and veterinary implications. We searched three databases (PubMed, Google Scholar, and Web of Science) from 1912 to June 30, 2023, resulting in 94 papers included in the qualitative analysis and 86 papers in the quantitative analysis. All identified field studies were conducted in endemic areas and investigated the presence of *Trypanosoma* and *Leishmania* parasites, DNA, or antigens in ticks. We recorded a pooled prevalence of Trypanosomatidae detection in ticks at 15.48 % [7.99–24.61 %], with significant variations depending on the year, detection method, and geographical area. Most of the infected tick species belonged to the genera *Amblyomma*, *Hyalomma*, *Ixodes*, and *Rhipicephalus*. Experimental laboratory work on transmission routes demonstrated potential vector competence in both the Argasidae and Ixodidae tick families. Although our systematic review and meta-analysis provide compelling evidence of the natural infection of ticks by Trypanosomatidae parasites, along with some evidence of non-traditional transmission routes, they do not offer conclusive evidence regarding the role of ticks as biological or mechanical vectors for Trypanosomatidae species of veterinary and medical interest. This highlights the urgent need for additional investigations to address this point.

## 1 Introduction

Ticks (Parasitiformes: Ixodida) are obligate hematophagous ectoparasites of all terrestrial vertebrate classes and are vectors or reservoirs of human and animal pathogenic virus, bacteria, protozoa and fungi [1]. Two tick families encompassing numerous genera hold considerable significance in public health and veterinary matters [2]. Currently, there are 996 known tick species worldwide, divided into three families: approximately 774 hard tick species (Ixodidae), around 221 soft tick species (Argasidae), and the monotypic family Nuttalliellidae, which exhibits features of both families [3,4]. The identification of tick fossils in amber has led to the description of two novel families: Deinocrotonidae and Khimairidae, alongside several unique genera, including *Compluriscututla*, *Cornupalpatum*, *Deinocroton*, and *Khimaira* [5]. Most protozoan pathogens carried by ticks that affect mammals of medical or veterinary interest are classified under the order Piroplasmida (e.g., *Babesia*, *Theileria*, and *Cytauxzoon*) or as haemogregarines within the *Hepatozoon* genus (Adeleorina: Hepatozoidae) [6].

The Trypanosomatidae family consists of unicellular eukaryotes, including pathogens of humans and animals from the genera *Trypanosoma* and *Leishmania,* as well as *Endotrypanum* and *Porcisia*. The life cycle of these organisms is primarily characterized by the involvement of arthropod vectors from the Hemiptera and Diptera orders, with some trypanosomes involving vectors from the Siphonoptera order, and fish trypanosomes from the Arhynchobdellida (Hirudinidae, leech) order. Two subspecies of *Trypanosoma brucei* (i.e., *Trypanosoma brucei gambiense* and *T. brucei rhodesiense*), along with *T. cruzi* and *T. rangeli*, as well as at least 23 species of *Leishmania*, including the recently described *Leishmania (Mundinia) chancei,* are pathogenic to humans. These pathogens cause human African trypanosomiasis (sleeping sickness), Chagas disease, and cutaneous, mucocutaneous, or visceral leishmaniasis [[7], [8], [9], [10], [11], [12], [13]]. These diseases also affect domestic, feral, and wild animals. Canine visceral leishmaniasis is primarily caused by *L. infantum* infection, with occasional cases attributed to *L. donovani* or *L. tropica* [14]. *Trypanosoma congolense*, *T. evansi,* *T. b. brucei*, *T. vivax*, *T. simiae*, *T. suis*, *T. theileri*, and, more rarely, *T. godfreyi* infect livestock. *Trypanosoma equiperdum* infects equids during mating [15,16]. Additionally, *Trypanosoma theileri*, considered non-pathogenic to humans, is found in cattle, buffalo, and antelope worldwide [17]. It has been infrequently linked to disease resembling nagana in specific cases involving a calf [18], cattle [19], or a cow [20], and it can cause illness in cattle under severe stress due to concurrent diseases or poor nutrition [21]. *Trypanosoma caninum* was described in 2014 as a new species infecting dogs, typically in asymptomatic cases with low humoral immune responses [22]. Furthermore, *T. lewisi*, a parasite of *Rattus*, is also an opportunistic parasite in humans and shares common vertebrate hosts with *T. cruzi* [23]. Altogether, more than 30 million people are infected, and over 48 million cattle are at risk of contracting animal trypanosomiasis in Africa, resulting in approximately 3 million cattle deaths annually [24].

Since the early 20th century, the transmission of protozoan parasites from *Leishmania* and *Trypanosoma* has been investigated, and more recently, molecular biology techniques (e.g., next-generation sequencing) have been employed to revisit this hypothesis [25,26]. There remains an ongoing debate within the scientific community regarding the ability of ticks to transmit Trypanosomatidae parasites of medical or veterinary interest. To provide an updated perspective on this debate, we conducted a systematic review and meta-analysis with the following objectives: (i) to gather published field and experimental data on the detection of Trypanosomatidae with medical and veterinary relevance in ticks, including *T. theileri*, *T. lewisi*, and *T. caninum*, and to assess their capacity to act as vectors for these pathogens; and (ii) to explore factors associated with the transmission of these pathogens by ticks.

## 2 Materials and methods

### 2.1 Protocol and registration

The current study was conducted following the Preferred Reporting Items for Systematic Reviews and Meta-analyses (PRISMA) guidelines [27]**.** The protocol was neither registered nor published. The checklist for meta-analysis is provided as Supplementary Material (Check List S1).

### 2.2 Information source

The systematic screening of existing literature was performed using PubMed, Google Scholar, and Web of Science (WOS) databases. Publish or Perish (Harzing.com), a software that retrieves and analyses academic citations was used to retrieve relevant articles from Google Scholar.

### 2.3 Search

A set of keywords was used: “Trypanosomatidae” and all species of “*Trypanosoma*” or “*Leishmania*”, known to be pathogenic for humans or animals and having medical or veterinary interest, including *T. theleiri*, *T. lewisi* and *T. caninum*, in combination with “ticks” “Ixodidae”, “Argasidae”, and selected tick genus (e.g., “*Rhipicephalus*”, “*Hyalomma*”, “*Ornithodoros*”). Database search was done between January 1900 and June 30^,^ 2023, without language restrictions. Articles reporting “Trypanosomatidae” in ticks by direct examination (e.g., culture, microscopy, immunohistochemistry “IHC”) or molecular method (e.g., conventional Polymerase chain reaction “PCR”, real-time PCR “RT-PCR”, capillary or next generation sequencing “NGS”) were included in the study. We exclude studies not in the scope of our study and reporting, i.e., those whose scope and objective are “Vaccine”, “Virus”, “Drug”, “Treat”, “Pest”, “Acaricide”, “Immunology”, “Serology”, “Serum”, “ELISA”, “Antigen”.

### 2.4 Eligibility criteria and study selection

We conducted the review in accordance with the current recommendations established in 2015 and reported our findings following the PRISMA guidelines, specifically addressing the remarks for “biological” meta-analyses [28]. Two authors independently performed a preliminary review of the articles by examining their titles and abstracts. Articles selected by at least one reviewer were retrieved and duplicated (same bibliographical record) papers were excluded. The two reviewers then performed a second selection based on full-text analysis, resolving any disagreements through discussion with a third reviewer. Studies were considered eligible based on established inclusion or exclusion criteria.

The selection of eligible articles was based on the following criteria: (1) articles addressing with the detection or transmission of Trypanosomatidae parasites of medical or veterinarian interest, including *T. theleiri*, *T. lewisi* and *T. caninum*, in ticks; (2) field or experimental studies; (3) no restrictions on host origin; and (4) no language restriction. The exclusion criteria included (1) certain literature categories (letters, books, and reviews); (2) studies that did not focus on ticks as vectors for Trypanosomatidae (e.g., cellular immune responses of ticks, host immunogenetic influences on tick resistance); (3) studies that did not focus on Trypanosomatidae of medical or veterinary interest; (4) studies lacking information on tick collection locations and origins; and (5) studies that did not specify the identity of tick and Trypanosomatidae species (6) studies not stating the sample size and the number of positive cases where excluded from the meta analysis.

### 2.5 Data collection

We utilized a pre-existing template to extract data from the selected articles, which two of the authors compiled into a Microsoft Excel® spreadsheet. The collected information included the authors' names, publication year, study subregion/country, tick family and species, identification methods, number of tested and positive tick samples, and detection methods.

### 2.6 Quality assessment

The quality of the selected publications was assessed using the Grading of Recommendations Assessment, Development, and Evaluation (GRADE) methodology [29]. The total score for each article was calculated based on the following seven criteria: (1) the tick family and species provided; (2) the identified Trypanosomatidae species; (3) the detection method used; (4) the number of tested ticks reported; (5) the number of positive ticks reported; (6) information on the prevalence of Trypanosomatidae parasites in ticks; and (7) the identity of the host from which the positive tick(s) originated. Items 1–5 were scored at 2 points each, while items 6 and 7 were scored at 1 point each. Based on the total score, each publication was classified as high quality (score = 8–12), medium quality (score = 5–7), or poor quality (score = 0–4).

### 2.7 Statistical analysis

For data related to field studies, we conducted a meta-analysis of proportions [30] using the ‘meta’ and ‘metafor’ packages in R software version 4.3.1. A Freeman-Tukey transformation with double arc sine (PFT) was applied to convert the proportions before meta-analysis. This transformation standardizes and stabilizes the distribution variance [31] (dat<-escalc(measure="PFT", xi=xi, ni=ni, data=dat). Due to the high heterogeneity expected in the meta-analysis, a random-effects model was employed to combine the overall effect size perform and subgroup analyses. Cochrane statistics *I*^*2*^ and *Q* (expressed as *X*^*2*^ and *P*, respectively) were used to assess and quantify heterogeneity. An *I*^*2*^ value < 50 % indicates low heterogeneity, whereas *I*^*2*^ value > 50 % signifies high heterogeneity. Meta-analysis statistics were visualized using Forest plots. The Egger test and Funnel plots were utilized to assess publication bias, and results underwent stability analysis, which evalues the impact of excluding data from any single article on the results of the remaining studies.

We also carried out subgroup analyses of potential risk factors, including sampling year (before 2000, 2001–2009, 2010–2019, 2020 and after), continent (Europe, Asia, Africa, South America, and Iceland), host family (Canidae, Bovidae, etc.), tick genus, tick species, detection methods, parasite type, and the location in the tick's organs. Additionally we performed a meta-regression using the studied parameters as covariates to address possible sources of heterogeneity.

Regarding the experimental studies, many papers lack quantitative data, such as the number of ticks used in the research. To address this, we performed a meta-analysis using semi-quantitative data, specifically the detection or absence of pathogens in ticks (0: absence, 1: presence). Through this methodology, we analyzed the detection of pathogens in ticks after blood feeding, the transmission of Trypanosomatidae to uninfected hosts during blood feeding, infection following the injection of infected tick materials, vertical transmission, and transstadial passage. Statistical tests were conducted, and subgroup analyses of associated factors were performed based on tick family, tick species, donor host, receiving host, and parasite family and species.

## 3 Results

### 3.1 Selection process overview: curating datasets for systematic review

**Fig. 1:**
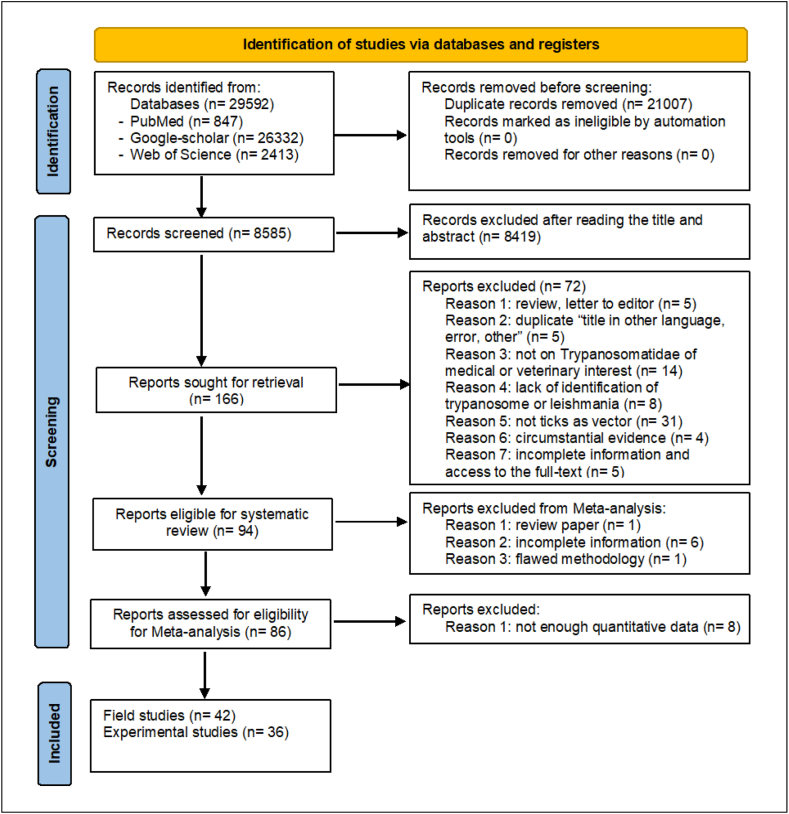
PRISMA flowchart illustrating the process of the systematic review and meta-analysis.

A total of 29,592 articles were retrieved during the systematic search on multiple databases, including PubMed, Google Scholar, and WOS, (Fig. 1). After the removal of duplicate paper (n = 21,007) and ineligible papers (n = 8,423), 166 published studies were selected (last updated June 30, 2023). After analysis of their titles, abstracts, and detailed contents, out of those 166 articles, 94 satisfied the eligibility criteria to be included in the systematic review (84 in English and 10 in other languages *i. e* Portuguese, Russian …), 86 articles were eligible for the meta-analysis, of which 49 dealt with field studies and 37 with experimental studies. In addition, 46 focused on the transmission of *Leishmania*, while 40 are focused on *Trypanosoma*. Full-text Portable Document Format (PDF) files not freely accessible online were obtained through the French Development Research Institute (IRD) library. Publications in French, German, Spanish, Portuguese, Russian, and Turkish languages were handled by authors and/or native language colleagues.

### 3.2 Chronology of publications on ticks as vectors of Trypanosomatidae infecting human and animals of veterinary interests

**Fig. 2:**
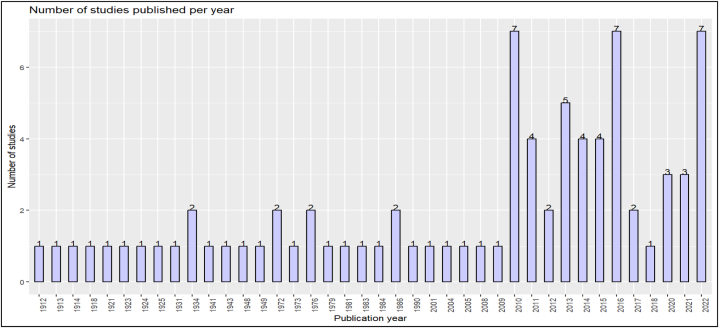
Temporal distribution of collected and selected scientific papers.

The phylum Arthropoda hosts both monoxenous and dixenous Trypanosomatidae [32]. Sand flies (Diptera; Phlebotominae) are known biological vectors of *Leishmania*, with some exceptions [33]. Other biological vectors of *Trypanosoma* include Diptera (e.g., tsetse flies, tabanids, and *Stomoxys*), Hemiptera (e.g., triatomine ‘reduviid’ bugs), and Siphonaptera (e.g., fleas) [34,35]. Although most Trypanosomatidae colonize members of the class Insecta, presumably monoxenous trypanosomatids also develop in ticks (class Arachnida) [[36], [37], [38], [39], [40], [41], [42], [43], [44], [45], [46], [47], [48], [49], [50], [51], [52], [53], [54], [55], [56], [57], [58], [59], [60], [61], [62], [63], [64], [65], [66], [67], [68], [69], [70], [71], [72], [73], [74], [75], [76], [77], [78], [79], [80], [81], [82], [83], [84], [85], [86], [87], [88], [89], [90], [91], [92], [93], [94], [95], [96], [97], [98], [99], [100], [101], [102], [103], [104], [105], [106], [107], [108], [109], [110], [111], [112], [113], [114], [115], [116], [117], [118], [119], [120], [121], [122], [123], [124], [125], [126], [127], [128], [129], [130], [131], [132], [133], [134], [135], [136], [137], [138], [139], [140], [141]]. Since the late 19th and early 20th centuries, field trials and experiments have been conducted to detect these parasites in ticks (Fig. 2). The ability of ticks to transmit *Leishmania* parasites has been documented for quite some time [37]. In recent years, advancements in molecular detection methods for Trypanosomatidae DNA in ticks have renewed interest in the role of ticks in the transmission of both *Trypanosoma* and *Leishmania*. Specifically, since 2010, the number of studies investigating this topic has increased significantly.

#### 3.2.1 Ticks as vectors of *Trypanosoma:* historical aspects

The hypothesis that ticks may act as reservoirs and/or vectors for human and animal trypanosomes has been investigated since the early 20th century [[38], [39], [40], [41], [42], [43], [44], [45], [46], [47], [48], [49], [50], [51], [52]]. In 1972, Hoare reviewed existing data on ticks as potential vectors and concluded that ticks could not transmit trypanosomes due to the absence of infective trypomastigote forms [34]. However, subsequent research documented the presence of various trypanosome developmental stages in ticks, including amastigotes, sphaeromastigotes, epimastigotes, and trypomastigotes, suggesting a more complex relationship between ticks and trypanosomes [53,54].

#### 3.2.2 *Leishmania*: a parasite transmitted by ticks?

The hypothesis that ticks may contribute to the transmission of *Leishmania* emerged in the 20th century. Early experiments suggested that ticks could act as vectors for *Leishmania* species responsible for human and canine kala-azar (*L. donovani/L. infantum* complex) in the Mediterranean region. The observation of *Leishmania* surviving for extended periods in the tick gut raised questions about the possibility of direct transmission [55]. However, this hypothesis has been challenged, with conflicting evidence regarding the involvement of ticks in the *L. infantum* transmission cycle [56]. In the mid-1980s, further doubt was cast on this theory, although one study demonstrated transmission during a tick's blood meal [57]. Subsequent field and laboratory studies have detected *Leishmania* RNA or DNA in various tick organs, including salivary glands and ovaries, across different developmental stages (larvae, nymphs, and adults), providing additional support for the potential role of ticks in *Leishmania* transmission [[58], [59], [60], [61], [62], [63], [64], [65], [66], [67], [68], [69], [70], [71], [72], [73], [74], [75], [76], [77], [78], [79], [80], [81], [82]].

### 3.3 Observations of Trypanosomatidae of medical and veterinary interest in field-collected tick specimens

As detailed in the following sections, numerous field studies have been conducted to investigate the prevalence of pathogenic Trypanosomatidae in ticks. Detection of the parasite has been achieved through various methods, including direct examination (e.g., culture, microscopy), molecular tools such as Polymerase Chain Reaction (PCR), quantitative PCR (qPCR), capillary sequencing, and next-generation sequencing, as well as immunological techniques like immunochromatography.

#### 3.3.1 *Trypanosoma* carriage and prevalence

Traces of *Trypanosoma* species of medical and veterinary importance have been reported in ticks, including *T. cruzi*, *T. vivax*, *T. evansi*, *T. theileri*, *T. theileri*-like, *T. congolense*, and *T. caninum* [49,[83], [84], [85], [86], [87], [88], [89], [90], [91], [92], [93], [94], [95], [96]]. All these studies focused on hard tick species (Ixodidae). Most of the positive tick species belonged to four genera: *Amblyomma*, *Hyalomma*, *Ixodes*, and *Rhipicephalus*, including *A. cajennense* [88,95], *A. longirostrum* [49], *A. variegatum* [83], *H. detritum* [93], *H. marginatum* [85], *I. ricinus* [91,93], *R. (Boophilus) microplus* [86,92,95], *R. (Boophilus)* spp. [90], *R. sanguineus* [87,93], *R. sanguineus* s.l. [94,96], and *Rhipicephalus* sp [89]. Conversely, other species tested negative for *Trypanosoma* spp. (Table 1) [89,90,[93], [94], [95], [96], [97]]. Ticks collected from dogs and cattle were more frequently infected by *Trypanosoma* compared to those collected from other domestic animals (e.g., sheep, goats, camels) or wild animals (e.g., foxes, boars).

• *Trypanosoma cruzi*

**Table 1:** Detection of Trypanosoma species of medical and veterinary importance in field-collected ticks.

Species	Sampling year/*Publication year§*	*Trypansoma* species	Tick infection status (P/N)&	Localization in the tick	Detection method	Host	Country	Continent	Refs
*A. cajennense*	2013	*T. cruzi*	N	H-Gr	Microscopy, PCR	*Canis lupus*	Brazil	South America	[97]
	*1905*	*T. vivax*	N	H	Microscopy	*Bos taurus*	Cuba	South America	[95]
	*1905*	*T. vivax*	P	G	Microscopy	*Bos taurus*	Cuba	South America	[95]
	*2021*	*T. vivax*	P	Gr	PCR, Sequencing	buffalos	Brazil	South America	[88]
*A. longirostrum*	*1941*	*T. cruzi*	P	NS	NS	Cercolabidae (*Arboreal porcupine*)	Venezuela	South America	[49]
*Amblyomma* spp.	2013–2019	*T. cruzi*	N	Gr	Metagenomics	**∗** Wild ungulates Carnivores	Kenya	Africa	[89]
*A. tigrinum*	2013	*T. cruzi*	N	Gr	PCR, Sequencing	*Canis lupus*	Chile	South America	[94]
*A. variegatum*	*2016*	*T. congolense*	P	Gr	Parasitological analysis, PCR	Cattle	Nigeria	Africa	[83]
*Haemaphysalis* spp.	2017–2018	*T. evansi*	N	Gr	PCR, Sequencing	Cattle	India	Asia	[90]
*H. detritum*	1982	*T. theileri*	P	H	Microscopy	*Bos taurus*; *Canis lupus*	Algeria	Africa	[93]
*H. dromedarii*	2015–2016	*T. evansi*	N	Gr	PCR	**∗∗**Wild ruminants	Tunisia	Africa	[96]
*H. excavatum*	2015–2016	*T. evansi*	N	Gr	PCR	**∗∗**Wild ruminants	Tunisia	Africa	[96]
	1982	*T. theileri; T. evansi*	N	H	Microscopy	*Bos taurus*; *Canis lupus*	Algeria	Africa	[93]
*H. lusitanicum*	1982	*T. theileri; T. evansi*	N	H	Microscopy	*Bos taurus*; *Canis lupus*	Algeria	Africa	[93]
*H. marginatum*	2015–2016	*T. evansi*	N	Gr	PCR	**∗∗**Wild ruminants	Tunisia	Africa	[96]
	*1990*	*T. theileri*	P	H	Microscopy	*Bos taurus*	Portugal	Europe	[85]
	1982	*T. theileri; T. evansi*	N	H	Microscopy	*Bos taurus*; *Canis lupus*	Algeria	Africa	[93]
*Hyalomma* spp.	2017–2018	*T. evansi*	N	Gr	PCR, Sequencing	Cattle	India	Asia	[90]
*I. ricinus*	2013	*T. caninum*	P	Gr	Culture, PCR, Sequencing	Vegetation	Slovakia	Europe	[91]
	1979–1982	*T. theileri*	P	H	Microscopy	*Bos taurus*; *Canis lupus*	Switzerland	Europe	[93]
	2015–2016	*T. evansi*	N	Gr	PCR	**∗∗**Wild ruminants	Tunisia	Africa	[96]
*R. (Boophilus) microplus*	NS	*T. theileri-like*	P	H	ND	*Bos taurus*	Brazil	South America	[92]
	*1905*	*T. vivax*	N	H	Microscopy	*Bos taurus*	Cuba	South America	[95]
	*1905*	*T. vivax*	P	G	Microscopy	*Bos taurus*	Cuba	South America	[95]
	NS	*T. vivax*	P	Gr	PCR	*Bos taurus*	Venezuela	South America	[86]
	*2021*	*T. vivax*	P	Gr	PCR, Sequencing	buffalo	Brazil	South America	[88]
*R. (Boophilus)* spp.	2017–2018	*T. evansi*	P	Gr	PCR, Sequencing	Cattle	India	Asia	[90]
*R. bursa*	2015–2016	*T. evansi*	N	Gr	PCR	**∗∗**Wild ruminants	Tunisia	Africa	[96]
*R. sanguineus*	2013	*T. cruzi*	N	H-Gr	Microscopy, PCR	*Canis lupus*	Brazil	South America	[97]
	1982	*T. evansi*	P	H	Microscopy	*Bos taurus*, *Canis lupus*	Algeria	Africa	[93]
	*2013*	*T. evansi*	P	Gr	PCR	*Canis lupus*	Brazil	South America	[87]
	*2013*	*T. vivax*	P	Gr	PCR	*Canis lupus*	Brazil	South America	[87]
*R. sanguineus* s.l	2013	*T. cruzi*	P	Gr	PCR, Sequencing	*Canis lupus*	Chile	South America	[94]
	2015–2016	*T. evansi*	P	Gr	PCR	Wild ruminants	Tunisia	Africa	[96]
*Rhipicephalus* spp.	2013–2019	*T. cruzi*	P	Gr	Metagenomics	**∗ ∗ ∗** Wild ungulates Carnivores Regular domestic	Kenya	Africa	[89]
*R. turanicus*	1982	*T. theileri; T. evansi*	N	H	Microscopy	*Bos taurus*; *Canis lupus*	Algeria	Africa	[93]

**§:** When the sampling date was not specified in the publication, the year of publication was added in *italics*; **&** information on positivity is available in the data collection sheet in the supplementary material**; P:** positive; **N:** negative; **F:** faeces**; G:** gut**; Gr:** whole body**; H:** hemolymph**; O:** ovaries**; SG:** salivary glands; **PCR:** conventional Polymerase chain reaction, **Sequencing:** Capillary sanger sequencing. **∗ Wild ungulates [**Black rhinoceros (*Diceros bicornis*), white rhinoceros (*Ceratotherium simum*), buffalo (*Syncerus caffer*), elephant (*Loxodonta africana*), giraffe (*Giraffa camelopardalis*), Grévy's zebra (*Equus grevyi*), plains zebra (*Equus quagga*), hartebeest (*Alcelaphus buselaphus*), impala (*Aepyceros melampus*)**]**; **Carnivores [**leopard (*Panthera pardus*), lion (*Panthera leo*), spotted hyena (*Crocuta crocuta*), wild dog (*Lycaon pictus*)**]**; **Regular domestic [**farmed Boran (*Bos indicus*) and cattle (*Bos taurus*)**]. ∗∗Wild ruminants:** Scimitar-horned oryx; Addax antelope; Barbary red deer; Dorcas gazelle. **∗∗∗ Wild ungulates [**plains zebra (*Equus quagga*)**]**; **Carnivores [**lion (*Panthera leo*), spotted hyena (*Crocuta crocuta*)**]**.

Since the 1940s, the prevalence of *T. cruzi* in field-collected ticks has remained uncertain. The presence of *T. cruzi* in *A. longirostrum*, an ectoparasite of porcupines, was documented during a survey of ectoparasites conducted in the state of Yaracuy, Venezuela [49]. Between 2013 and 2015, ticks collected from 148 dogs in the urban area of Campo Grande, Mato Grosso do Sul, Brazil, revealed that *R. sanguineus* and *A. cajennense* were not infected by *T. cruzi* [97]. More recently, the presence of *T. cruzi* was documented in dogs and their ectoparasites in a rural area of central Chile, where 57 % of blood samples were infected by *T. cruzi*, and 5.4 % of ticks tested positive by PCR. Specifically, *R. sanguineus* s.l. (5/82) was positive, while all *A. tigrinum* specimens (0/11) were negative [94]. The same year, a study focused on using ticks as xenosurveillance sentinels to monitor circulating pathogens in the Kenyan drylands. Ticks were collected from wild ungulates, carnivores, domestic animals, and Boran cattle and screened using metagenomics. *T. cruzi* DNA was detected in 3 out of 46 (6.5 %) pools of *Rhipicephalus* spp., but not in *Amblyomma* spp [89]. However, the examination of the sequences could not definitively confirm the presence of *T. cruzi*.

• *Trypanosoma vivax*

*Trypanosoma vivax* infects wild and domestic ungulates and is transmitted mechanically via tabanids and other blood-sucking insects in the Americas [88]. An analysis of the gut contents of *A. cajennense* (Fabricius) sensu stricto (s.s.) and *R. (Boophilus) microplus* (Canestrini) (Acari: Ixodidae), collected from cattle (*Bos taurus*) on two farms in Cuba, revealed the presence of living *T. vivax* forms 96 h after repletion, though not in the hemolymph [95]. Of the 285 *R. (Boophilus) microplus* ticks collected from cattle in livestock areas of Merida, Venezuela, 7.7 % tested positive using PCR [86]. That same year, an examination of 63 *R. sanguineus* ticks collected from dogs in Campo Grande, Brazil, revealed the presence of *Trypanosoma* spp. via PCR, with 15 (23.8 %) testing positive for *T. vivax* [87]. Lastly, in a search for *T. vivax* in *A. cajennense* s.s. and *R. (Boophilus) microplus* collected from cattle, 6.25 % (3/48) of *A. cajennense* s.s. and 4.5 % (2/45) of *R. (Boophilus) microplus* were positive. The sequences obtained were 99 % identical to those of bovine *T. vivax* from northeastern Brazil [88].

• *Trypanosoma evansi*

*Trypanosoma evansi* is primarily transmitted by tabanid flies and *Stomoxys* spp. It affects a wide range of hosts, including livestock, camelids, equids, carnivores, rodents, and humans, producing variable clinical symptoms depending on the host [96]. In a study on the seasonal dynamics of *R. sanguineus* in dogs in urban areas of western Algeria, hemolymph smears were examined for potential microorganisms. Five out of 250 *R. sanguineus* ticks (four females and one male) were found to be infected with trypanosomes, representing 2 % of the total. Two infected females showed pathological reactions (globular bodies and milky hemolymph) related to the infection. Based on the observed form and small size of the protozoan, it was hypothesized to be *T. evansi* (referred to as *T. berberum* in the manuscript), a parasite of camelids that causes deadly trypanosomiasis in dogs [93]. This hypothesis was further supported by a report that 15 out of 63 dogs in Campo Grande, Mato Grosso do Sul, Brazil, tested positive for *T. vivax* and 7 for *T. evansi* using PCR [87]. In 2021, 0.2 % of the 352 ticks collected from the environment and wild ruminants in Tunisia were also reported positive for *T. evansi* by PCR [96]. Finally, in India, the sequencing of 240 pooled tick-DNA samples collected from two regions revealed no prevalence of *T. evansi* in Karnataka but a prevalence of 8.3 % in Kerala [90].

• *Trypanosoma theileri* and *T. theileri-like*

Multiple studies have documented the presence of trypanosomes in tick hemolymph [36,53,84,93,98]. Most of these studies identified *T. theileri*, which is typically transmitted by tabanid flies [99]. *T. theileri* was first reported in field-collected ticks in a 1979 study conducted in Switzerland, where 0.19 % (5 out of 2501) of *I.* *ricinus* were found to carry trypanosomes morphologically similar to *T. theileri* [84]. Between 1979 and 1982, the presence of *T. theileri* in the hemolymph of ticks from Switzerland and Algeria was confirmed, with 0.24 % (37/1570) of *H. detritum* and *I. ricinus* ticks collected from bovines testing positive [93]. In 2008, Martins reported the presence of Trypanosomatidae epimastigote forms in the hemolymph of the cattle tick *R. (Boophilus) microplus* in Rio Grande do Sul, Southern Brazil, with microscopic examination suggesting *T. theileri* [92].

• *Trypanosoma congolense*

Animal trypanosomosis is a complex disease caused by one or more species of pathogenic trypanosomes, including *T. congolense*. Clinical symptoms are characterized by intermittent fever, parasitemia, anemia, lymphadenopathy, jaundice, progressive emaciation, weakness, and reduced productivity [100]. Trypanosomes transmitted by tsetse flies (*Glossina* spp.) are responsible for animal African trypanosomosis in sub-Saharan Africa [101]. In 2016, trypanosomes were detected in engorged adult *A. variegatum* collected from cattle in the Unguwan Rimi and Kaduna state areas of northwestern Nigeria. Of the 33 samples examined microscopically, 14 tested positive for *T. congolense*, representing a prevalence rate of 42.4 %. On the first day of parasitemia follow-up, 10 (30.3 %) samples were microscopically positive for *T. congolense*, while 23 (69.7 %) were negative [83].

• *Trypanosoma caninum*

*Trypanosoma caninum*, the most recently identified species within the Trypanosomatidae family, has been reported to infect dogs in Brazil [22]. Since its description, 67 cases of natural infection in dogs have been documented in areas where canine visceral leishmaniasis is endemic [22,102]. In Slovakia, a novel trypanosome was isolated and partially characterized from *I. ricinus*. Sequence analysis suggests that this trypanosome, referred to as *Trypanosoma* sp. Bratislava1, is a new species closely related to several trypanosomes isolated from or detected in ticks in South America and Asia, including *T. caninum* from Brazil [91]the most recent species within the Trypanosomatidae family, was reported to infect dogs in Brazil [22]. Despite its recent identification, 67 cases of natural infection in dogs have been documented in areas where canine visceral leishmaniosis is endemic [22,102]. In Slovakia, a novel trypanosome was isolated and partially characterized from *I. ricinus*. The resulting sequences support this trypanosome, referred as *Trypanosoma* sp. Bratislava1, as a new species closely related to several trypanosomes isolated from, or detected in, ticks in South America and Asia, including *T. caninum* isolated in Brazil [91].

#### 3.3.2 *Leishmania* prevalence in field collected ticks

The presence of *Leishmania* in ticks is well documented, including species such as *L. infantum/L. donovani*, *L. major*, *L. chagasi* (syn. *L. infantum*), *L. braziliensis*, *L. guyanensis*, and *L. martiniquensis* [64,[66], [67], [68], [69], [70], [71], [72], [73],[75], [76], [77], [78], [79], [80], [81],^97^,[103], [104], [105], [106], [107], [108], [109], [110], [111]]. Most field studies focus on ticks from domestic animals in urban areas, particularly *R. sanguineus* [60,64,[66], [67], [68],^70^,^71^,^75^,[79], [80], [81],^87^,[103], [104], [105], [106],^108^,^109^,^111^]. Ticks collected from more wild ecosystems have highlighted the diversity of tick species positive for the presence of *Leishmania*, including *A. sabanerae* [110], *Amblyomma* spp. [76], *A. tigrinum* [71], *A. variegatum* [69], *Hyalomma aegyptium* and *H. dromedarii* [73], *Ixodes ricinus* [75,77,79,107,109], *I.* spp. [109], *I. ventalloi* [75,109], *R. (Boophilus) microplus* [69,76,110], *R. pusillus* [75,109], *R. sanguineus* s.l., and *R. turanicus* [73]. (Table 2).

• *Leishmania infantum and L. chagasi (Syn* L. *infantum)*

**Table 2:** Detection of Leishmania species of medical and veterinary importance in field-collected ticks.

Species	Sampling year/Publication year§	Leishmania species	Tick status(P/N)&	Localization in the tick	Detection method In tick	Host	Detection method in the host	Country	Continent	Ref
*A. cajennense*	2013	*L. chagasi* (Syn *L. infantum*)	N	H-Gr	Microscopy, PCR	*Canis lupus*	ND	Brazil	South America	[97]
*A. ovale*	2008	*L. infantum*	N	Gr	qPCR	*Canis lupus*	IFAT	Brazil	South America	[63]
*A. sabanerae*	2022	*Leishmania* sp.	P	Gr	qPCR	*Pecari tajacu*; *Chelonoidis denticulata*	ND	Peru	South America	[112]
*Amblyomma* spp.	2012	*L. guyanensis*	P	Gr	PCR; HRM-PCR	*Tapirus terrestris*; *Pecari tajacu*	ND	Peru	South America	[76]
*A. tigrinum*	2010–2013	*Leishmania* sp.	P	Gr	PCR	*Pseudalopex griseus*	PCR, qPCR	Argentina	South America	[71]
*A. variegatum*	2014–2015	*L. martiniquensis*	P	Gr	HT-qPCR	Cattle	ND	Guadeloupe	Island	[69]
*D. marginatus*	2007–2008	*L. infantum*	N	Gr	PCR	Wild boars	ND	Italy	Europe	[113]
	2007	*L. infantum*	N	Gr	qPCR	*Canis lupus*	ELISA, PCR	Italy	Europe	[78]
	2014	*Leishmania* sp.	N	Gr	PCR	Human	ND	Turkey	Asia	[114]
*Ha. longicornis*	2012	*L. infantum*	N	Gr	PCR	Sheep, cattle and dog	ND	China	Asia	[115]
*Ha. parva*	2014	*Leishmania* sp.	N	Gr	PCR	Human	ND	Turkey	Asia	[114]
*Ha. punctata*	2014	*Leishmania* sp.	N	Gr	PCR	Human	ND	Turkey	Asia	[114]
*Ha. sulcata*	2007–2008	*L. infantum*	N	Gr	PCR	Sheep and Goat	ND	Italy	Europe	[113]
	2014	*Leishmania* sp.	N	Gr	PCR	Human	ND	Turkey	Asia	[114]
*H. aegyptium*	2015–2018	*L. infantum*	P	Gr	PCR; Sequencing	*Testudo graeca*; *Camelus dromedarius*	ND	Israel	Asia	[72]
	2014	*Leishmania* sp.	N	Gr	PCR	Human	ND	Turkey	Asia	[114]
*H. dromedarii*	2015–2018	*L. infantum*	P	Gr	PCR, Sequencing	*Testudo graeca*; *Camelus dromedarius*	ND	Israel	Asia	[72]
*H. excavatum*	2014	*Leishmania* sp.	N	Gr	PCR	Human	ND	Turkey	Asia	[114]
*H. marginatum*	2007–2008	*L. infantum*	N	Gr	PCR	Cattle	ND	Italy	Europe	[113]
	2014	*Leishmania* sp.	N	Gr	PCR	Human	ND	Turkey	Asia	[114]
*Hyalomma* spp.	2014	*Leishmania* sp.	N	Gr	PCR	Human	ND	Turkey	Asia	[114]
*I. ricinus*	2007	*L. infantum*	P	Gr	qPCR	*Canis lupus*	ELISA, PCR	Italy	Europe	[78]
	2007–2008; 2010	*L. infantum*	P	Gr	PCR	Dog, horse, cat, bovine, human	ND	Italy	Europe	[77]
	2011 and 2013	*L. infantum*	P	Gr	PCR	*Felis catus*	ND	Italy	Europe	[75]
*I. ricinus*	2012–2013	*L. infantum*	P	Gr	qPCR	*Felis catus*	PCR, qPCR	Italy	Europe	[109]
	2010	*L. infantum*	P	Gr	PCR; Sequencing	$Flagging	ND	Italy	Europe	[107]
	2014	*Leishmania* sp.	P	Gr	PCR	Human	ND	Turkey	Asia	[114]
*Ixodes* spp.	2011 and 2013	*L. infantum*	N	Gr	PCR	*Felis catus*	ND	Italy	Europe	[75]
	2012–2013	*L. infantum*	P	Gr	qPCR	*Felis catus*	PCR, qPCR	Italy	Europe	[109]
*I. ventalloi*	2011 and 2013	*L. infantum*	P	Gr	PCR	*Felis catus*	ND	Italy	Europe	[75]
	2012–2013	*L. infantum*	P	Gr	qPCR	*Felis catus*	PCR, qPCR	Italy	Europe	[109]
*R. (Boophilus) microplus*	2012	*L. guyanensis*	P	Gr	PCR; HRM-PCR	*Tapirus terrestris*; *Pecari tajacu*	ND	Peru	South America	[76]
	2012	*L. infantum*	N	Gr	PCR	Sheep, cattle and dogs	ND	China	Asia	[115]
		*Leishmania* sp.	P	Gr	qPCR	*Pecari tajacu; Chelonoidis denticulata*	ND	Peru	South America	[110]
	2014–2015	*L. martiniquensis*	P	Gr	HT-qPCR	Cattle	ND	Guadeloupe	Island	[69]
	2014–2015	*L. martiniquensis*	P	Gr	HT-qPCR	Cattle	ND	Martinique	Island	[69]
*R. bursa*	2014	*Leishmania sp.*	N	Gr	PCR	Human	ND	Turkey	Asia	[114]
	2007–2008	*L. infantum*	N	Gr	PCR	Sheep, goat, cattle, horse, deer	ND	Italy	Europe	[113]
*R. pusillus*	2011 and 2013	*L. infantum*	P	Gr	PCR	*Felis catus*	ND	Italy	Europe	[75]
	2007–2008	*L. infantum*	N	Gr	PCR	Hedgehog	ND	Italy	Europe	[113]
	2012–2013	*L. infantum*	P	Gr	qPCR	*Felis catus*	PCR, qPCR	Italy	Europe	[109]
*R. sanguineus*	2012–2013	*L. infantum*	P	Gr	qPCR	*Felis catus*	PCR, qPCR	Italy	Europe	[109]
	2016–2017	*L. major*	P	Gr	PCR, Sequencing	*Rhombomys opimus*; *Nesokia indica*	ND	Iran	Asia	[103]
	2013	*L. chagasi* (syn *L. infantum*)	P	H-Gr	Microscopy, PCR	*Canis lupus*	ND	Brazil	South America	[97]
	NS	*L. braziliensis*	P	Gr	PCR, qPCR	*Canis lupus*	ND	Brazil	South America	[66]
	2007	*L. infantum*	P	Gr	PCR; qPCR	*Canis lupus*	IFAT, PCR, qPCR	Brazil	South America	[105]
	2007	*L. infantum*	P	Gr-SG	PCR; qPCR; sequencing	*Canis lupus*	ND	Italy	Europe	[64]
	2008	*L. infantum*	P	Gr-SG	PCR; qPCR; sequencing	*Canis lupus*	ND	Brazil	South America	[64]
	2007–2008	*L. infantum*	P	Gr	PCR	*Canis lupus*	ELISA	Brazil	South America	[108]
	2007–2008	*L. infantum*	N	Gr	PCR	*Canis lupus*	ND	Italy	Europe	[113]
	2008–2009	*L. infantum*	P	Gr	PCR, RT-PCR, Sequencing	*Canis lupus*	ELISA, PCR	Brazil	South America	[60]
*R. sanguineus*	2007	*L. infantum*	P	Gr	qPCR	*Canis lupus*	ELISA, PCR	Italy	Europe	[78]
	2006–2007	*L. infantum*	P	Gr	qPCR	*Canis lupus*	ELISA, PCR	Italy	Europe	[79]
	2011–2012	*L. infantum*	N	NS	Infestation	*Canis lupus*	IFAT, ELISA	Brazil	South America	[116]
	2013	*L. infantum*	P	Gr	PCR, qPCR, seuqencing	*Canis lupus*	PCR, qPCR	Brazil	South America	[67]
	2012	*L. infantum*	P	Gr	PCR	*Canis lupus*	IFAT, ELISA	Brazil	South America	[59]
	2013	*L. infantum*	P	Gr	PCR, Sequencing	*Canis lupus*	PCR, Sequencing	Brazil	South America	[68]
	2011	*L. infantum*	P	G	PCR, RFLP, seuqencing, Parasit culture	*Canis lupus*	DPP, IFAT, ELISA, PCR	Brazil	South America	[70]
	2011	*L. infantum*	P	SG	PCR, RFLP, Sequencing, Parasit culture	*Canis lupus*	DPP, IFAT, ELISA, PCR	Brazil	South America	[70]
	2011 and 2013	*L. infantum*	P	Gr	PCR	*Felis catus*	ND	Italy	Europe	[75]
	2002	*Leishmania* sp.	P	G	Microscopy	*Canis lupus*	IFAT	Brazil	South America	[111]
	2009	*Leishmania* sp.	N	NS	present of tick or no	*Canis lupus*	IFAT	Brazil	South America	[74]
	2012–2014	*Leishmania* sp.	N	Gr	qPCR	*Canis lupus*	qPCR	China	Asia	[117]
	2015	*Leishmania* sp.	P	G; O; SG	IHC, qPCR, IHC	*Canis lupus*	ND	Brazil	South America	[81]
	2016	*Leishmania* sp.	P	G; O; SG	IHC, qPCR, IHC	*Canis lupus*	ND	Brazil	South America	[80]
	1934	£L. kala azar	P	NS	NS	Human	NS	France	Europe	[106]
	2008	*L. infantum*	N	Gr	qPCR	*Canis lupus*	IFAT	Brazil	South America	[63]
*R. sanguineus s.l.*	NS	*L. infantum*	P	Gr	PCR, Sequencing	$Flagging & ∗Animals	ND	Israel	Asia	[72]
*R. turanicus*	2007–2008	*L. infantum*	N	Gr	PCR	Sheep and Goat	ND	Italy	Europe	[113]
	2014	*Leishmania* sp.	N	Gr	PCR	Human	ND	Turkey	Asia	[114]
	2022	*L. infantum*	P	Gr	PCR, Sequencing	$Flagging & ∗Animals	ND	Israel	Asia	[72]

**§:** When the sampling date was not specified in the publication, the year of publication was added in *italics*; **&** information on positivity is available in the data collection sheet in the supplementary material**; NS**: Not specified; **P:** positive; **N:** negative; **F:** faeces**; G:** gut**; Gr:** crushing**; H:** hemolymph**; O:** ovaries**; SG:** salivary glands; **PCR:** conventional Polymerase chain reaction, **RFLP:** restriction fragment length polymorphism; **qPCR:** real time PCR; **RT-PCR**: reverse transcriptase PCR; **HRM-PCR**: High Resolution Melting PCR; **HT-qPCR**: High-throughput microfluidic qPCR; **Sequencing:** Capillary sanger sequencing, **IFAT:** Indirect Fluorescent Antibody Test; **ELISA:** Enzyme Linked Immunosorbent Assay; **DPP:** rapid immunochromatographic test; **IHC**: immunohistochemistry **∗ Animals:** dogs (*Canis familiaris*); Tortoise (*Testudo graeca*); hedgehogs (*Erinaceus concolor*); badger. ^***£***^**L. kala azar:** refers to members of the *L. donovani* complex (*L. infantum* and *L. donovani*) without any other information on parasite typing at the time of the study. We therefore use the term proposed by the author. ^**$**^**Flagging**: refers to the use of a method to collect questing ticks.

The hypothesis that ticks could serve as vectors for *Leishmania* was first proposed in the early 20th century [118]. Domestic dogs play a central role as reservoirs of *L. infantum* in the peridomestic zoonotic transmission cycle. The brown dog tick, *R. sanguineus* (Latreille 1806), has been the subject of extensive research due to its prevalence among urban dogs [119]. The overall prevalence of *L. infantum* in *R. sanguineus* collected from dogs [59,60,64,67,70,78,79,105,120] or cats [75,109] ranges from 2.5 % to 70.3 % in Brazil and Italy. Significant L. *infantum* presence has been recorded in various *Rhipicephalus* species, including 9.2 % of *R. sanguineus* s.l. ticks collected by flagging (questing ticks) and 23.5 % of *R. turanicus* collected from dogs, hedgehogs, and tortoises in Israel [73]. Additionally, infections were reported in *R. pusillus* from cats in Italy, with 10.9 % and 17.6 % positivity. Studies have also detected *L. infantum* in *I. ricinus* (1.5 %–50 %) [75,77,79,107,109], *I. ventalloi* (19 % and 62 %) [75,109], *Ixodes* sp. (10.9 %) [98], *H.* *aegyptium* (38.7 %), and *H. dromedarii* (55.6 %) [73], collected from various hosts, including dogs, horses, cats, bovines, tortoises, camels, and humans in Italy and Israel. In most of these studies, *L. infantum* DNA was detected in whole ticks, and in some cases, specifically in the gut or salivary glands [64,70]. *Leishmania chagasi* (syn. *L. infantum*), which causes American visceral leishmaniasis, is widely distributed across Latin America [121]. It is believed that *L. infantum* was introduced to South America by the conquistadors' dogs [122]. In Campo Grande, Mato Grosso do Sul, Brazil, two tick species, *R. sanguineus* and *A. cajennense*, were collected from dogs in urban areas. Tick samples from 36 dogs tested positive for *L. infantum*, all of which were *R. sanguineus* [97].

• *Leishmania major*

One *R.* *sanguineus* nymph collected from a rodent in the Segzi Plain, Esfahan Province, Iran, was tested positive for *L. major* [103].

• *Leishmania braziliensis*

American cutaneous leishmaniasis (ACL) is primarily caused by *L. braziliensis*. The challenges in controlling ACL may be linked to the disease's complex epidemiology, which involves various vector species, including ticks. Two rural areas in Pernambuco, northeastern Brazil, where ACL is endemic, were investigated for canine ectoparasites. Genomic DNA was extracted from 75 *R*. *sanguineus* ticks, 32 of which (42.67 %) tested positive for *L. braziliensis* using both conventional PCR and real-time PCR (RT-qPCR) [66].

• *Leishmania guyanensis*

*Leishmania guyanensis* is a causative agent of American tegumentary leishmaniasis. In 2017, DNA belonging to the *Viannia* subgenus was detected in 81 *R*. *(Boophilus) microplus* and *Amblyomma* ticks collected from three *Tapirus terrestris* and three *Pecari tajacu* in Madre de Dios, Peru, following amplification of kinetoplast DNA (kDNA) [55]. Additionally, *Leishmania (Viannia)* kDNA was detected in three *R. microplus* ticks collected from a *P. tajacu* hunted in the forests of Madre de Dios. High-Resolution Melting PCR (HRM-PCR) identified one positive sample with a kDNA melting curve compatible with *L. (V.) guyanensis* [76].

• *Leishmania martiniquensis*

*Leishmania martiniquensis* was first isolated in 1995, its taxonomical classification was established in 2002, and it was officially named in 2014 [11,[123], [124], [125]]. The vectors and reservoirs remain unidentified, although biting midges are suspected to be involved in the transmission of *Leishmania* species belonging to the *Mundinia* subgenus [123,126]. In 2020, a high-throughput microfluidic real-time PCR system designed for genus-level parasite screening was applied to 132 adult specimens of *Amblyomma variegatum* and 446 specimens of *R*. *microplus* collected in Guadeloupe and Martinique. It was found that 0.7 % of the *R. microplus* ticks from Martinique tested positive for *Leishmania* spp., with sequences identifying *L. martiniquensis* [69].

• Other/*unidentified Leishmania* parasites

In 2010, the presence of leishmania promastigote forms in ticks parasitizing domestic dogs was reported in the Municipality of São Vicente Férrer, located in the northern agreste of Pernambuco, Brazil [111]. In 2015 and 2016, leishmania was detected in the intestines, ovaries, and salivary glands of *R. sanguineus* from dogs in Brazil, using both immunohistochemistry (IHC) and real-time PCR. IHC revealed *Leishmania* in 98 % of intestines, 14 % of ovaries, and 8 % of salivary glands, while real-time PCR confirmed these organs as the most positively tested sites [80,81]. Additionally, *Leishmania* DNA, likely belonging to the *L. donovani* complex, was found in 11 out of 17 pools (64.7 %) of *A*. *tigrinum* ticks collected from eight foxes (six grey foxes, Pseudalopex griseus, and two culpeo foxes, P. culpaeus) in Argentine Patagonia [71]. Although sandflies are typically found 2000 km and 750 km north of the study area, this finding suggests either a wider distribution than currently believed or the persistence of *Leishmania* in another vector. In 2022, 95.7 % of R. (Boophilus) microplus and 90 % of *A. sabanerae* collected from *Pecari tajacu* and *Chelonoidis denticulata* in leishmaniasis-endemic zones of the Peruvian Amazon were found to carry *Leishmania*, with parasite loads of 34.1 and 5428.6 per arthropod, respectively [127].

### 3.4 Experimental infection and transmission of Trypanosomatidae by ticks

In the early 20th century, laboratory experiments were conducted to assess the vector competence of ticks for transmitting trypanosomes. Several studies demonstrated the presence of various *Trypanosoma* or *Leishmania* forms—including trypomastigotes, amastigotes, epimastigotes, promastigotes, and sphaeromastigotes—in both ixodid and argasid ticks. Transmission routes were investigated, including transmission through tick bites and blood meals, as well as alternative routes such as ingestion of infected ticks, transstadial passage (between different life stages of ticks and their hosts), and transovarial transmission (ovarian infection).

#### 3.4.1 Transmission of *Trypanosoma* following tick bites and feeding

The role of ticks as vectors of trypanosomes has been increasingly supported by recent studies, which provide additional evidence of the transmission of distinct trypanosome clades to various mammalian species by ticks [26]. Several reports document the detection of *Trypanosoma* in ticks after blood-feeding on an infected animal in experimental settings [40,43,45,46,128,129]. Trypanosomes of medical and veterinary interest have been detected in both hard and soft ticks from three genera (*Rhipicephalus*, *Hyalomma*, and *Ornithodoros*) and four species: *R. sanguineus*, *H. a*. *anatolicum*, *O. crossi*, and *O. lahorensis*. Experimental infections following a bite or blood meal have been reported for *T. cruzi*, *T. evansi*, and *T. theileri* [130].

• *Trypanosoma cruzi*

The earliest reports of dog infection by *T. cruzi*-infected *R. sanguineus* ticks date back to 1913 [40]. However, attempts to infect rats using infected soft ticks of the genus *Ornithodoros* were unsuccessful [41]. Several other studies have also failed to demonstrate the transmission of *T. cruzi* by soft ticks in laboratory settings, including *O. moubata*, *O. talaje*, and *O. turicata*.

• *Trypanosoma evansi*

Investigations into the transmission of *T*. *evansi* by ticks began in the early 20th century. Some studies suggested that the soft tick *Or*. *crossi*, infected with *T. evansi*, could transmit the parasite to non-infected animals after a blood meal [43,44,46]. However, in 1924, Yorke and Macfie did not confirm the transmission of this protozoan by *O. crossi* [45]. Similarly, in 1976, Taylor-Lewis reported the unsuccessful transmission of *T. evansi* by *H*. *dromedarii* to rodents after blood feeding [131]. More recently, Mahmoud et al. (2020) also failed to detect *T. evansi* DNA in uninfected rodents after blood feeding by infected *O. savignyi* [132].

• *Trypanosoma theileri and Trypanosoma theileri-like*

In 1981, a pioneering experimental study reported the inability of *H*. *anatolicum* carrying *T*. *theileri* to infect calves [54]. However, this initial observation was challenged by subsequent studies conducted a few years later [123,124].

#### 3.4.2 Transmission of *Leishmania* following tick bites and feeding

To date, sandflies (Phlebotominae) are recognized as the primary biological vectors of *Leishmania* [133], though the potential role of ticks, fleas, and leeches as secondary vectors has been suggested [61,134]. Five laboratory studies have attempted to investigate transmission after collecting ticks from vertebrates experimentally infected with *Leishmania* [37,56,57,82,135]. All experiments were conducted on hard ticks (Ixodidae), with only ticks from the *Rhipicephalus* genus involved. Ticks were fed on either dogs or rodents, and two species were used: *R*. *turanicus* and *R. sanguineus*. *Rhipicephalus. sanguineus* was the only species shown to be infected by *Leishmania*, specifically *L. infantum* and *Leishmania* sp [57,82].

• *Leishmania infantum*

The transmission of *L*. *infantum* by ticks was first reported in 1930 by Blanc and Caminopétros, who infected squirrels after exposure to *R*. *sanguineus* ticks infected with what they referred to as ‘Leishmania kala azar,’ which most likely corresponds to parasites of the *L. donovani* complex today [37]. However, this finding was not confirmed by later studies [51,131]. In contrast, a report of successful transmission to 14 hamsters by *R. sanguineus* infected with *L. infantum* was documented [82], though this transmission method has not been further supported by subsequent research [135].

#### 3.4.3 Host infection following ingestion of infected ticks or injection with *Trypanosoma*-infected tick materials

Documented infection by trypanosomes following the ingestion of an infected invertebrate suggests that trypanosomes may also be transmitted through the ingestion of infected ticks [34,137,138]. To date, nine studies on the Argasidae family and five on the Ixodidae family have investigated the transmission of trypanosomes in the laboratory, either through ingestion of infected ticks or by injection of infected material [41,42,45,47,48,[50], [51], [52], [53], [54],^98^,^128^,^129^,^131^]. Of these 14 studies, nine confirmed infections through the injection of infected tick material, while and only one confirmed infection through ingestion of infected ticks. However, drawing clear conclusions from these results remains challenging. Seven tick species were tested, *O. moubata, O. crossi, O. venezuelensis, O. turicata, O. furcosus, R. pulchellu*s, and *H. a. anatolicum excavatum*. Five *Trypanosoma* species were tested.

• *Trypanosoma cruzi*

Experimental transmission of *T. cruzi* through ingestion or injection of tick-infected material began in the 20th century. The injection of *O. moubata*-material infected with *T. cruzi* successfully caused rodent infection [41,42]. Later studies confirmed rodent infection by *T. cruzi*-infected *O. venezuelensis, O. turicata, O. furcosus,* and *O. moubata* ticks [47,48,50,52].

• *Trypanosoma theileri* and *T. theileri-like*

Pioneering studies conducted in 1973 documented the inability to infect rodents by injecting *T. theileri*-infected hard tick material collected from cattle [53]. In contrast to this observation, successful infections has been reported using *O. moubata* or *H. a. anatolicum* material infected with *T. theileri* [49,98,117,118].

• Other *Trypanosoma*

As early as 1924, Yorke and Macfie attempted to transmit *T. rhodesiense* to rodents by injecting them with material from infected *O. crossi* ticks [45]. In 1948, Packchanian was unsuccessful in infecting animals with *T. brucei* using similar protocol [51]. Finally, in 1976, Taylor-Lewis also failed to infect rodents by injecting material from *H. a. excavatum* ticks infected with *T. lewisi* [131].

#### 3.4.4 Infection after ingestion or injection with *Leishmania*-infected ticks material

Several studies have reported the presence of *L. infantum* in *R. sanguineus* and *I. ricinus* [60,64,70,79,104], as well as the transmission of other protozoa, such as *Hepatozoon canis*, to dogs after ingestion of ticks [139]. Six studies have experimentally investigated the transmission of *Leishmania* via the oral route and the infective potential of injecting material from infected ticks [[55], [56], [57],^61^,[63], [64], [65]]. Among these, five confirmed infections were documented in seven reports-four after injection and one after ingestion. All these studies focused exclusively on ticks from the Ixodidae family, specifically *R. sanguineus*, which were infected with various *Leishmania* species and fed on rodents or dogs.

• *Leishmania infantum and Leishmania chagasi (*Syn: *L. infantum)*

Experimental infection of *L. infantum* by injecting infected *R. sanguineus* material was reported in 2010 [64]**.** This finding was further supported by Coutinho and colleagues demonstrating infection with *L. chagasi (syn: L. infantum)* in rodent from infected dog ticks [61].

• *Leishmania*

In 1984, a study assessed infection by injecting material from crushed, infected R. sanguineus ticks into two hunting dogs, crossbred between a German Shepherd and a Labrador Retriever. Following the injection, *Leishmania* species were detected in the recipient dogs using several diagnostic methods, suggesting the possibility of infection through the injection of tick-infected material [57].

### 3.5 Transstadial transmission in ticks

The ability of a microorganism to survive the shedding process and persist for an extended period within the tick is crucial for successful transstadial transmission. For *T*. *thylacis* such transmission was documented in the tick *I. tasmani*, and then to the Australian short-nosed bandicoot (*Isoodon macrourus*) [140].

#### 3.5.1 Transstadial transmission of *Trypanosoma*

Transstadial passage of *T*. *theileri* in *H*. *a. anatolicum* ticks was reported in larvae and nymphs that fed on infected calves and tested positive after molting to the adult stage [54]. This finding was corroborated in more recent studies [129].

#### 3.5.2 Transstadial transmission of *Leishmania*

Transstadial transmission of *L*. *infantum* in *R*. *sanguineus* has been documented since the 1930s [37]. Recent studies have once again demonstrated the persistence of *L. infantum* kDNA in *R. sanguineus* nymphs and adults that fed on infected dogs before molting [74]. However, the presence of live parasites could not be confirmed through microscopic examination or culture from the infected tick material [74].

### 3.6 Transovarial passage

#### 3.6.1 Transovarial passage of *Trypanosoma*

The detection of flagellates and trypanosomes in tick ovaries was first reported in 1961 [53,98,141], leading to the proposal of transovarial transmission as a potential alternative route for maintaining the parasite in the wild. The earliest experimental reports of transovarial transmission of *Trypanosoma* in ticks date back to the early 1900s with *T. cruzi* [41], though further evidence was lacking for *O*. *moubata* or *Rhipicephalus* ticks [39,48]. Transovarial transmission was also investigated for *T. theileri* in *Hyalomma a. anatolicum*, but no conclusive evidence was found [41]. It wasn't until 2004 that a laboratory study demonstrated pathogenic trypanosomes being transmitted via tick ovaries [129]. No conclusive evidence of transovarial transmission of *T. evansi* in *R. sanguineus* has been reported [142].

#### 3.6.2 Transovarial passage of *Leishmania*

Recent reports have confirmed the transovarial transmission of *Leishmania* in *R*. *sanguineus*, with the first evidence of this mode of transmission emerging in the early 1980s [57]. Experimental studies have demonstrated the presence of *L. infantum* kDNA in *R. sanguineus* larvae four months after experimental infection of females [65,74,118]. However, controversies still exist [61,62], and the only experiment conducted with *L. major* produced no conclusive evidence [143].

### 3.7 Other circumstantial evidence

Significant associations between tick infestation prevalence and the presence of anti-*Leishmania* antibodies in dogs from endemic areas of canine leishmaniasis have been reported [63,116,[144], [145], [146]]. The prevalence of *R*. *sanguineus* infestation was significantly higher (p = 0.04) among seropositive dogs (38.5 %) compared to their seronegative counterparts (29.0 %). The probability of leishmania seropositivity was found to be 1.5 times higher in tick-infested dogs than in non-infested animals [61,140,141]. Conversely, the odds of infection did not significantly differ between non-infested and *R. sanguineus*-infested dogs in one study [116].

The near-perfect correlation between leishmania detection in dogs through fine-needle aspiration biopsy of the lymph nodes and the detection of leishmania in the tick's intestine via immunohistochemistry provides additional circumstantial evidence [147].

## 4 Meta-analyses results

### 4.1 Field detection

#### 4.1.1 Overview of the meta-analysis

**Fig. 3:**
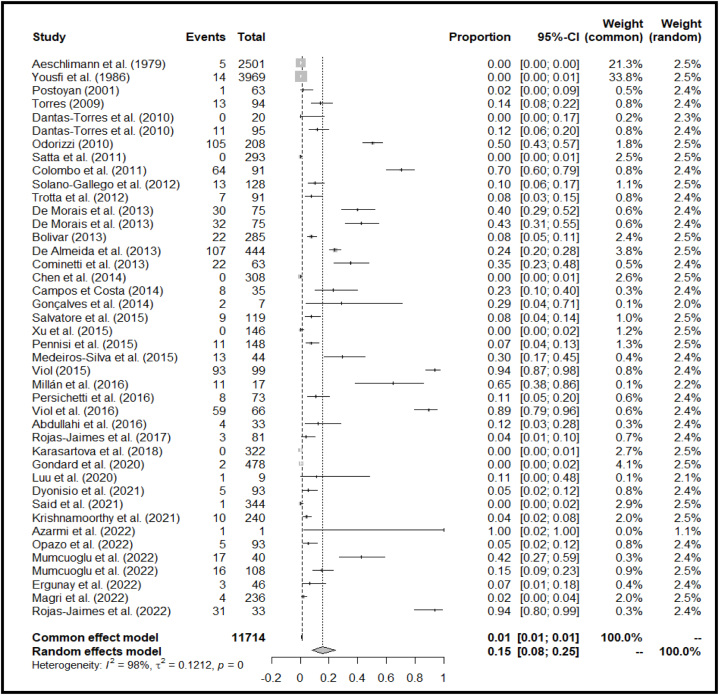
Forest plot showing the prevalence of Trypanosomatidae detection in field-collected ticks. The horizontal lines represent the 95 % confidence intervals, while the diamond indicates the pooled effect size.

**Fig. 4:**
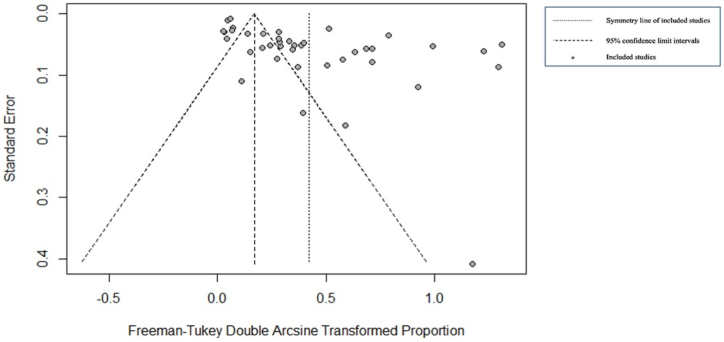
Funnel plot with 95 % confidence intervals for assessing publication bias.

For the field studies, we identified 42 publications for meta-analysis and meta-regression (Fig. 3). The use of a random-effects model was justified by the *P* and *I*^*2*^ statistics, which showed significantly high heterogeneity (*X*^*2*^ = 3551.8296 and *I*^*2*^ = 98.99 %, *p* = 0.0001; Fig. 3). We identified publication bias in the selected studies based on visual inspection of the graph's asymmetry (Fig. 4), which was confirmed by Egger's test (*t* = 4.15, *p* = 0.0002) (**Supplementary File**, Table S1). The elimination, one by one or simultaneously, of the four publications showing bias (Fig. 4) did not affect the cumulative prevalence recorded.

The 42 studies selected included approximately 12,000 ticks collected worldwide. However, in some publications, it was not possible to determine the exact number of ticks used for the experiment. Therefore, the data we used for our analysis was the number of pools. Our results depicted an overall cumulative prevalence for Trypanosomatidae detection of 15.48 % (95 % CI: 7.99–24.61 %) (Fig. 3).

We then examined factors associated with the detection of *Leishmania* and *Trypanosoma* having medical/veterinary interest in ticks. Factors examined included the sampling decade, study area (continent), animal host (family), tick genus or species, the detection method, parasite species, and location in the tick's organs (Table 3). We recorded high heterogeneity in all subgroups; therefore, the pooled seroprevalence estimate for each subgroup was calculated using the random-effects model.

**Table 3:** Pooled prevalence of parasite detection in ticks, categorized by sampling year, continent, host, tick genus/species, detection method, parasite genus/species, and localization.

Category	Variable	No. of studies	No. of tested	No. of positive	% [95 % CI]	Heterogeneity			Univariate meta regression		
						ꭕ2	*P*-value	*I*^*2*^ (%)	*P*-value	*R*^*2*^ (%)	*I*^*2*^-res (%)
Sampling year	2000 or before	2	6470	19	0.22 [0.16–0.45]	1.14	<0.0001	12.0	0.000∗∗∗	0.00	98.74
	2001–2010	5	480	130	12.19 [0.87–32.08]	126.37	<0.0001	96.8			
	2011–2019	23	3043	518	21.13 [7.7–23.77]	818.31	<0.0001	97.3			
	2020 or after	12	1721	96	11.65 [0.3–30.84]	289.5	<0.0001	96.2			
Continent	Africa	4	4392	22	2.44 [0.00–9.54]	23.24	<0.0001	85.8	0.0056∗∗	23.77	97.78
	Asia	7	1165	44	3.11 [0.00–20.83]	131.61	<0.0001	95.4			
	Europe	10	3693	69	4.36 [1.49–9.06]	158.52	<0.0001	94.3			
	Island	1	478	2	0.42 [0.01–1.26]	0.000	<0.0001	–			
	South America	20	1986	626	33.35 [14.27–37.83]	828.68	<0.0001	96.1			
Host (Family)	Several#	6	4894	43	2.83 [0.0–8.60]	80.46	<0.0001	93.8	0.0007∗∗	0.00	98.34
	Bovidae	6	1192	44	3.92 [1.20–7.85]	24.26	<0.0001	88.2			
	Canidae	20	2184	595	27.92 [14.51–43.57]	1071.56	<0.0001	98.2			
	Felidae	2	221	19	8.47 [5.07–12.60]	0.81	<0.0001	0.0			
	Other	7	722	57	21.23 [0.0–62.24]0	252.480	<0.0001	97.6			
Tick Genus	*Amblyomma*	10	328	27	8.78 [0–29.41]	100.12	<0.0001	91.0	0.0001∗∗∗	2.20	97.09
	*Dermacentor*	3	23	0	0.00 [0–5.25]	0.41	<0.0001	0.0			
	*Haemaphysalis*	4	360	00	0.00	1.58	<0.0001	0.0			
	*Hyalomma*	6	639	17	1.20 [0–14.55]	70.55	<0.0001	92.9			
	*Ixodes*	9	6906	42	1.91 [0–10.06]	71.41	<0.0001	88.8			
	*Rhipicephalus*	33	3271	668	19.06 [9.64–30.39]	1412.59	<0.0001	97.7			
Tick species	*A. cajennense*	1	131	0	0.00 [0–7.04]	3.86	<0.0001		0.0001∗∗∗	3.29	97.50
	*A. ovale*	1	10	0	0.00 [0–16.52]	0.00	<0.0001	–			
	*A. sabanerae*	1	10	9	90.00 [61.91–100]	0.00	<0.0001	0.0			
	*A. tigrinum*	2	28	0	24.86 [0–95.91]	16.89	<0.0001	–			
	*A. variegatum*	2	165	11	3.09 [0–24.26]	0.43	<0.0001	94.1			
	*Amblyomma* spp.	2	43	4	0.00 [0–0.64]	11.54	<0.0001	91.30.0			
	*D. marginatus*	3	21	0	0.00 [0–5.25]	0.59	<0.0001	0.0			
	*H. aegyptium*	2	32	12	32.69 [11.66–56.75]	0.46	<0.0001	0.0			
	*H. detritum*	1	1	1	100 [0–100]	0.00	<0.0001	–			
	*H. dromedarii*	2	45	5	16.56 [0–88.80]	17.10	<0.0001	94.2			
	*H. excavatum*	3	291	0	0.00	1.45	<0.0001	0.0			
	*H. lusitanicum*	1	3	0	0 [0–50]	0.00	<0.0001	–			
	*H. marginatum*	4	171	0	0.00	2.04	<0.0001	0.0			
	*Hyalomma* sp.	2	81	0	0.00 [0–2.36]	0.01	<0.0001	0.0			
	*Ha. longicornis*	1	308	0	0.00 [0–0.58]	0.00	<0.0001	–			
	*Ha. parva*	1	41	0	0.00 [0–4.15]	0.00	<0.0001	–			
	*Ha. punctata*	1	6	0	0.00 [0–26.80]	0.00	<0.0001	–			
	*Ha. sulcata*	2	4	0	0.00 [0–78.74]	0.07	<0.0001	0.0			
	*Haemaphysalis.* sp.	1	13	0	0.00 [0–12.82]	0.00	<0.0001	–			
	*I. ricinus*	8	6580	32	1.36 [0–10.39]	64.66	<0.0001	87.6			
	*I. ventalloi*	1	62	3	6.45 [1.42–14.19]	0.00	<0.0001	–			
	*Ixodes* sp.	1	5	0	0.00 [0–31.73]	0.00	<0.0001	–			
	*R. (Boophilus)*	6	1025	62	0.00 [0–3.92]	0.51	<0.0001	0.0			
	*R. bursa*	3	23	0	13.54 [0–47.12]	185.13	<0.0001	22.2			
	*R. pusillus*	2	18	3	17.65 [2.57–39.97]	0.02	<0.0001	0.0			
	*R. sanguineus*	21	2222	583	25.15 [11.67–41.19]	1092.57	<0.0001	97.9			
	*R. turanicus*	4	158	10	2.48 [0–1467]	23.70	<0.0001	87.3			
	*Rhipicephalus* sp.	1	43	3	6.98 [0.9–16.93]	0.00	<0.0001	–			
Detection method	Microscopy	3	6533	20	0.1 [0.0–0.23]	3.47	<0.0001	42.4	0.000∗∗∗	5.37	98.84
	Molecular	39	5181	743	17.55 [9.31–27.44]	1937.17	<0.0001	98.1			
Parasite genus	*Leishmania*	30	4569	719	18.87 [9.12–30.75]	1642.76	<0.0001	98.294.6	0.0046∗∗	7.10	98.88
	*Trypanosoma*	12	7739	93	4.62 [1.14–9.79]	203.03	<0.0001				
Parasite species	*L. braziliensis*	1	75	32	42.67 [31.64–64.66]	0.00	<0.0001	–	0.000∗∗∗	9.25	98.97
	*L. chagas (*syn *L. infantum)*	1	444	107	24.10 [20.23–28.19]	0.00	<0.0001	–			
	*L. guyanensis*	1	81	3	12.12 [2.79–25.84]	0.00	<0.0001	–			
	*L. infantum*	18	2124	323	15.44 [7.18–25.89]	679.35	<0.0001	97.5			
	*L. major*	1	1	1	100 [0–100]	0.08	<0.0001	–			
	*L. martiniquensis*	1	578	2	0.35 [0–1.04]	0.00	<0.0001	–			
	*Leishmania* sp.	6	683	194	51.82 [8.42–93.57]	0.00	<0.0001	94.4			
	*T. caninum*	1	9	1	11.11 [0–41.77]	0.00	<0.0001	–			
	*T. congolense*	1	33	4	3.70 [0.47–9.18]	0.00	<0.0001	–			
	*T. cruzi*	3	583	8	2.39 [0–10.25]	895.52	<0.0001	91.2			
	*T. evansi*	4	892	38	6.44 [0–23.33]	13.00	<0.0001	95.9			
	*T. theileri*	2	6220	14	0.20 [0.12–0.36]	73.71	<0.0001	0.0			
	*T. vivax*	3	441	42	10.88 [2.91–22.73]	22.82	<0.0001	84.6			
Localization in ticks	H	2	6470	19	0.29 [0.16–0.45]	1.14	<0.0001	12.0	0.000∗∗∗	0.00	98.89
	G	3	209	164	74.32 [28.48–99.99]	312.09	<0.0001	97.3			
	GR	35	4532	478	13.45 [6.62–21.87]	1234.08	<0.0001	97.2			
	GR-SG	1	95	11	11.58 [5.82–18.88]	0.00	<0.0001	–			
	H-GR	1	444	107	24.10 [20.23–28.19]	0.00	<0.0001	–			
	SG	2	110	35	31.79 [23.31–40.91]	0.00	<0.0001	0.0			
	O	2	165	74	44.48 [37.27–52.53]	0.67	<0.0001	0.0			

**G:** gut**; Gr:** whole body**; H:** hemolymph**; O:** ovaries**; SG:** salivary glands ∗0.05; ∗∗0.01; ∗∗∗0.001, # In these studies, various tick species were allowed to feed on diverse infected host (Swiss mice, Meadow voles, Rats, Mice, Rabbits, Camel, Dog, Guinea pig or White rat). In these papers, it was not possible to retrieve the information on the host origin of the ticks tested.

#### 4.1.2 Detection method

Molecular biology yielded the highest detection rates, reaching 17.55 % (with a confidence interval of 9.31–27.44 %), surpassing the rates detected by microscopic methods, which stand at a mere 0.1 % (confidence interval: 0–0.23 %). Despite the specificity of microscopy in identifying Trypanosomatidae [16,148] they demand skilled personnel for accurate parasite detection. The efficacy of these methods varies with the type of sample and generally falls short of the sensitivity and specificity offered by PCR and cell culture methods [149]. Due to their superior sensitivity and specificity, molecular techniques are regarded as more effective [16,150].

#### 4.1.3 Temporal analysis

There were statistically significant differences across the collection decade (Table 3). The highest annual prevalence recorded was 14.92 % (95 % CI: 7.7–23.77 %, 518/3043) between 2011 and 2019, and the lowest at 0.29 % (95 % CI: 0.16–0.45 %, 19/6470) was reported before the 2000s. This increasing prevalence trend since 2000 is likely due to advances in the performance of molecular detection methods [64,130] and/or the impacts of climate change on vector populations and behaviors [151,152].

#### 4.1.4 Tick's geographical origin and host genus

We also assessed differences according to geographical origin (continent) (Table 3). The highest prevalence, 25.17 % (95 % CI: 14.27–37.83 %, 626/1986) was recorded in South America, while the lowest was in Africa (*p* = 0.0056). The cumulative prevalence statistics by host animal on which ticks were collected, displayed a significant difference (p = 0.0012) between animal hosts, with the highest cumulative prevalence being on Canidae (27.92 % [14.51–43.57 %], 595/2184).

#### 4.1.5 Tick's genus

The prevalence of tick infection significantly varies according to the tick's taxonomic status (*p* = 0.0001), from 19.06 % (CI: 9.64–30.39 %, 668/3271) in *Rhipicephalu*s to 8.78 % (95 % CI: 0–29.41 %, 27/328) in *Amblyomma* or 1.91 % and 1.20 % in *Ixodes* and *Hyalomma*, respectively, to 0.0 % in *Dermacentor* and *Haemaphysalis* (Table 3).

#### 4.1.6 Detection method, genus and tick's organs

We recorded statistical differences according to the detection method (p > 0.05). The prevalence was higher at 17.55 % (95 % CI: 9.31, 27.44) with molecular methods and lowest at 0.1 % (95 % CI: 0–0.23) using microscopy. Moreover, the infection rate was significantly higher *(p* = 0.0046) at 18.87 % [9.12–30.75] for *Leishmania* than for *Trypanosoma* at 4.62 % [1.14–9.79]. Infection rate also varied significantly (p = 0.000) by organ in ticks: the highest rate is 74.32 % (95 % CI: 28.24–99.99, 164/209) in the digestive tract, followed by 44.48 % [37.27–52.53] in the ovaries, 31.79 % [23.31–40.91 %] in the salivary glands, and the lowest rate of infection is 0.29 % [95 % CI: 0.16–0.45 %] in the hemolymph (Table 3).

### 4.2 Experimental studies

For the experimental studies, 37 publications were selected. Data were extracted from these research papers. Quantitative data such, as the number of ticks used for experimental infections, were not considered in the meta-analysis. They were transformed into semi-quantitative ones (presence or absence of parasites following experimental infection or transmission). Since no heterogeneity was detected in the data set, the common-effect model was chosen for the meta-analysis.

#### 4.2.1 Ingestion after blood feeding on an infected host

The meta-analysis was performed on 36 studies. The analysis showed the presence of parasites in ticks after their blood meal in 91 % [72–100 %] of studies (Supplementary File, Fig. S1). The factors associated with the detection of *Leishmania* and *Trypanosoma* in the blood meal were analyzed taking into account each actor involved in the transmission cycle, tick, animal host and parasite (Table 4). No heterogeneity was detected in these subgroups. Rate estimates pooled for each subgroup were calculated using a common-effects model.

• Tick's family and genus

**Table 4:** Pooled rate of parasite detection in ticks from experimental transmission studies.

**Acquisition of pathogens via tick blood feeding**										
**Category**	**Variable**	**No. of studies**	**No. of positive**	**% [95** **% CI]**	**Heterogeneity**			**Univariate meta regression**		
					**ꭕ2**	***P*-value**	***I***^***2***^**(%)**	***P*-value**	***R***^***2***^**(%)**	***I***^***2***^**-res (%)**
Ticks Family	Argasidae	16	16	100 [81.65–100]	0.00	0.9966	0.0	0.0976	0.00	0.00
	Ixodidae	21	15	78.01 [48.91–98.34]	15.86	0.9966	0.0			
Ticks Genus	*Amblyoma*	1	1	100 [0–100]	0.00	0.9938	–	0.3905	0.00	0.00
	*Hyalomma*	4	3	82.18 [16.67–100]	2.78	0.9938	0.0			
	*Ornithodoros*	15	15	100 [80.68–100]	0.00	0.9938	0.0			
	*Rhipicephalus*	16	11	74.57 [40.89–98.60]	12.72	0.9938	0.0			
Ticks species	*A. americanum*	1	1	100 [0–100]	0.00	0.9989	–	0.6118	0.00	0.00
	*B. decoloratus*	1	1	100 [0–100]	0.00	0.9989	–			
	*D. andersoni*	2	2	100 [21.62–100]	0.00	0.9989	0.0			
	*H.* a*. anatolicum*	3	2	72.14 [2.45–100]	2.47	0.9989	18.9			
	*H.* a*. excavatum*	1	1	100 [0–100]	0.00	0.9989	–			
	*H. dromaderii*	1	1	100 [0–100]	0.00	0.9989	–			
	*H. impressum*	1	1	100 [0–100]	0.00	0.9989	–			
	*O. amblus*	1	1	100 [0–100]	0.00	0.9989	–			
	*O. crossi*	4	4	100 [48.72–100]	0.00	0.9989	0.0			
	*O. furcosis*	1	1	100 [0–100]	0.00	0.9989	–			
	*O. hermsi*	2	0	0 [0–78.74]	0.00	0.9989	0.0			
	*O. lahorensis*	2	2	100 [21.26–100]	0.00	0.9989	0.0			
	*O. moubata*	7	7	100 [65.46–100]	0.00	0.9989	0.0			
	*O. parkeri*	1	1	100 [0–100]	0.00	0.9989	–			
	*O. savignyi*	1	1	100 [0–100]	0.00	0.9989	–			
	*O. talaje*	1	0	0 [0–100]	0.00	0.9989	–			
	*O. turanicus*	2	2	100 [21.62–100]	0.00	0.9989	0.0			
	*O. venzualensis*	1	1	100 [0–100]	0.00	0.9989	–			
	*R. sanguneus*	13	11	91.99 [58.62–100]	6.26	0.9989	0.0			
	*R. sanguneuss.l.*	2	0	0 [0–78.74]	0.00	0.9989	0.0			
	*R. pulchellus*	1	1	100 [0–100]	0.00	0.9989	–			
Parasite genus	*Leishmania*	14	9	69.10 [62.64–97.17]	11.90	0.9974	0.0	0.0794	0.00	0.00
	*Trypanosoma*	22	21	99.11 [80.73–100]	3.53	0.9974	0.0			
Parasite Species	*L. donovani*	1	1	100 [0–100]	0.00	0.9928	–	0.7192	0.00	0.00
	*L. chagasi (syn L.infantum)*	2	2	100 [21.26–100]	0.00	0.9928	0.0			
	*L. infantum*	5	4	87.58 [29.26–100]	2.96	0.9928	0.0			
	*L. kala azar*^*£*^	4	1	17.82 [0–83.24]	2.78	0.9928	0.0			
	*L. major*	1	1	100 [0–100]	0.00	0.9928	–			
	*Leishmania* sp.	1	1	100 [0–100]	0.00	0.9928	–			
	*T. bruci*	1	1	100 [0–100]	0.00	0.9928	–			
	*T. cruzi*	8	8	100 [68.71–100]	0.00	0.9928	0.0			
	*T. evansi*	7	6	92.94 [46.20–100]	3.17	0.9928	0.0			
	*T. gambiense*	1	1	100 [0–100]	0.00	0.9928	–			
	*T. lewisi*	2	2	100 [21.26–100]	0.00	0.9928	0.0			
	*T. rhodesciense*	2	2	100 [21.26–100]	0.00	0.9928	0.0			
	*T. theileri*	4	3	82.18 [16.67–100]	2.78	0.9928	0.0			
	*T. theileri like*	1	1	100 [0–100]	0.00	0.9928	–			
Donor host family	Bovidae	4	3	82.18 [16.67–100]	2.78	0.9471	0.0	0.9455	0.00	0.00
	Canidae	13	9	75.34 [37.81–99.71]	10.25	0.9471	0.0			
	Camelidae	2	2	100 [21.26–100]	0.00	0.9471	0.0			
	Rodentia	15	14	93.73 [63.97–100]	42.76	0.9471	0.0			
	ND	2	2	100 [21.26–100]	0.00	0.9471	0.0			
	Other	1	1	100 [0–100]	0.00	0.9471	–			
	Ticks	1	1	100 [0–100]	0.00	0.9471	–			
Infection via the injection of ticks infected material										

Ticks Family	Argasidae	9	7	85.25 [41.62–100]	5.76	0.6062	0.0	0.2884	0.00	0.00
	Ixodidae	13	7	55.23 [18.49–89.66]	11.96	0.6062	0.0			
Ticks Genus	*Hyalomma*	4	2	50.0 [0–100]	3.70	0.5378	18.9	0.5954	0.00	0.00
	*Ornithodoros*	9	8	85.25 [41.62–100]	5.76	0.5378	0.0			
	*Rhipicephalus*	7	4	59.68 [11.06–99.18]	6.34	0.5378	5.4			
Ticks species	*B. decoloratus*	1	0	0 [0–100]	0.00	0.8803	–	0.2229	0.00	0.00
	*D. andersoni*	2	0	0 [0–78.74]	0.00	0.8803	0.0			
	*H. a. anatolicum*	3	2	72.14 [2.45–100	2.47	0.8803	18.9			
	*H. a. excavatum*	1	0	0 [0–100]	0.00	0.8803	–			
	*H. dromaderii*	1	0	0 [0–100]	0.00	0.8803	–			
	*H. impressum*	1	0	0 [0–100]	0.00	0.8803	–			
	*O. amblus*	1	1	100 [0–100]	0.00	0.8803	–			
	*O. crossi*	1	0	0 [0–100]	0.00	0.8803	–			
	*O. furcosus*	1	1	100 [0–100]	0.00	0.8803	–			
	*O. hermsi*	2	0	0 [0–78.74]	0.00	0.8803	0.0			
	*O. moubata*	4	4	100 [48.72–100]	0.00	0.8803	0.0			
	*O. perkeri*	1	1	100 [0–100]	0.00	0.8803	–			
	*O. talaje*	1	0	0 [0–100]	0.00	0.8803	–			
	*O. turanicus*	1	0	0 [0–100]	0.00	0.8803	–			
	*O. turicata*	2	1	50.0 [0–100]	1.85	0.8803	46.0			
	*O. venzualensis*	1	1	100 [0–100]	0.00	0.8803	–			
	*R. pulchellus*	1	0	0 [0–100]	0.00	0.8803	–			
	*R. sanguineus*	6	5	90.82 [38.83–100]	3.08	0.8803	0.0			
	*R. sanguineus s.I.*	1	0	0 [0–100]	0.00	0.8803	–			
Parasite genus	*Leishmania*	7	5	78.10 [27.34–100]	5.29	0.5774	0.0	0.7666	0.00	0.00
	*Trypanosoma*	14	9	69.10 [32.64–97.17]	11.9	0.5774	0.0			
Donor host Family	Bovidae	4	2	50.0 [0–100]	3.70	0.5180	18.9	0.7557	0.00	0.00
	Camelidae	1	1	100 [0–100]	0.00	0.5180	–			
	Canidae	5	4	87.58 [29.26–100]	2.96	0.5180	0.0			
	Rodentia	11	8	68.26 [27.33–98.66]	9.42	0.5180	0.0			
Receiver host Family	Bovidae	3	2	72.14 [2.45–100]	2.47	0.5387	18.9	0.5494	0.00	0.00
	Canidae	1	1	100 [0–100]	0.00	0.5387	–			
	Other	1	0	0 [0–100]	0.00	0.5387	–			
	Rodentia	15	11	63.49 [28.31–93.43]	13.32	0.5387	0.0			
	Ticks	2	2	100 [21.26–100]	0.00	0.5387	0.0			
Parasite species	*L. chagasi (Syn* L. *infantum)*	1	1	100 [0–100]	0.00	0.8397	–	0.2614	0.00	0.00
	*L. infantum*	1	1	100 [0–100]	0.00	0.8397	–			
	*L. kala azar*^*£*^	4	2	50.0 [0–100]	3.70	0.8397	18.9			
	*Leishmania* sp.	1	1	100 [0–100]	0.00	0.8397	–			
	*T. brucei*	1	0	0 [0–100]	0.00	0.8397	–			
	*T. cruzi*	6	6	100 [61.37–100]	0.00	0.8397	0.0			
	*T. evansi*	1	0	0 [0–100]	0.00	0.8397	–			
	*T. lewisi*	1	0	0 [0–100]	0.00	0.8397	–			
	*T. rhodeseinse*	1	0	0 [0–100]	0.00	0.8397	–			
	*T. theileri*	4	3	82.18 [16.76–100]	2.78	0.8397	0.0			
	*T. theileri like*	1	0	0 [0–100]	0.00	0.8397	–			
Transmission through blood feeding										

Ticks Family	Argasidae	10	3	23.71 [0–66.41]	7.77	0.5314	0.9	0.4962	0.00	0.00
	Ixodidae	11	5	34.33 [0.0–94]	10.09	0.5314	0.0			
Ticks Genus	*Hyalomma*	4	2	50.0 [0–100]	3.70	0.4677	18.9	0.7745	0.00	0.00
	*Ornithodoros*	10	3	23.71 [0–66.41]	7.77	0.4677	0.0			
	*Rhipicephalus*	7	3	40.32 [0.48–88.94]	6.32	0.4677	5.4			
Parasite genus	*Leishmania*	5	2	36.51 [0–92.81]	4.44	0.9952	9.9	0.5011	0.00	0.00
	*Trypanosoma*	16	8	33.22 [5.25–67.40]	13.88	0.9952	0.0			
Donnor host family	Bovidae	4	2	50.0 [0–100]	3.70	0.9017	18.9	**0.0405∗**	0.00	0.00
	Camelidae	2	2	100 [21.26–100]	0.00	0.9017	0.0			
	Canidae	7	4	59.68 [11.06–99.18]	6.34	0.9017	5.4			
	Rodentia	8	0	0 [0–31.29]	0.00	0.9017	0.0			
Receiver host family	Bovidae	3	2	72.14 [2.45–100]	2.47	0.8461	18.9	0.0678	0.00	0.00
	Canidae	3	2	72.14 [2.45–100]	2.47	0.8461	18.9			
	Other	2	2	100 [21.26–100]	0.00	0.8461	0.0			
	Rodentia	13	4	8.01 [0–41.38]	6.26	0.8461	0.0			
Ticks species	*B. decoloratus*	1	0	0 [0–100]	0.00	0.6280	–	0.6077	0.00	0.00
	*D. andersoni*	1	0	0 [0–100]	0.00	0.6280	–			
	*H. a. anatolicum*	3	2	72.14 [2.45–100]	2.47	0.6280	18.9			
	*H. a. excavatum*	1	0	0 [0–100]	0.00	0.6280	–			
	*H. dromaderii*	1	0	0 [0–100]	0.00	0.6280	–			
	*H. impressum*	1	0	0 [0–100]	0.00	0.6280	–			
	*O. crossi*	4	3	82.18 [16.67–100]	2.78	0.6280	0.0			
	*O. hermsi*	1	0	0 [0–100]	0.00	0.6280	–			
	*O. lahorensis*	2	2	100 [21.26–100]	0.00	0.6280	0.0			
	*O. moubata*	3	0	0 [0–61.92]	0.00	0.6280	0.0			
	*O. savigny*	1	0	0 [0–100]	0.00	0.6280	–			
	*O. talaje*	1	0	0 [0–100]	0.00	0.6280	–			
	*O. turanicus*	1	0	0 [0–100]	0.00	0.6280	–			
	*O. turicata*	1	0	0 [0–100]	0.00	0.6280	–			
	*O. venzualensis*	1	0	0 [0–100]	0.00	0.6280	–			
	*R. pulchellus*	1	0	0 [0–100]	0.00	0.6280	–			
	*R. sanguineus*	6	3	50.0 [2.78–97.22]	5.55	0.6280	9.9			
Parasite species	*L. infantum*	2	1	50.0 [0–100]	1.85	0.5302	46.0	0.5529	0.00	0.00
	*L. kala azar*^*£*^	2	0	0 [0–78.74]	0.00	0.5302	0.0			
	*Leishmania* sp.	1	1	100 [0–100]	0.00	0.5302	–			
	*T. cruzi*	6	1	9.18 [0–61.17]	3.08	0.5302	0.0			
	*T. evansi*	6	3	50.0 [2.78–97.22]	5.55	0.5302	9.9			
	*T. lewisi*	1	0	0 [0–100]	0.00	0.5302	–			
	*T. theileri*	3	1	27.86 [0–97.55]	2.47	0.5302	18.9			
	*T. theileri* like	1	1	100 [0–100]	0.00	0.5302	–			
Vertical transmission										

Ticks Family	Argasidae	1	0	0 [0–100]	0.00	0.4606	–	0.2809	0.00	0.00
	Ixodidae	12	7	61.27 [22.41–94.58]	10; 79	0.4606	0.0			
Ticks Genus	*Hyalomma*	2	1	50.0 [0–100]	1.85	0.3787	46.0	0.5422	0.00	0.00
	*Ornithodoros*	1	0	0 [0–100]	0.00	0.3787	–			
	*Rhipicephalus*	10	6	63.49 [21.05–97.58]	8.88	0.3787	0.0			
Ticks species	*H. a. anatolicum*	2	1	50.0 [0–100]	1.85	0.4143	46.0	0.4394	0.00	0.00
	*O. moubata*	1	0	0 [0–100]	0.00	0.4143	–			
	*R. pulchellus*	1	0	0 [0–100]	0.00	0.4143	–			
	*R. sanguineus*	9	7	72.14 [27.07–100]	7.40	0.4143	0.0			
Parasite genus	*Leishmania*	8	7	82.18 [35.16–100]	5.55	0.6668	0.0	0.0634	0.00	0.00
	*Trypanosoma*	5	1	12.42 [0–70.74]	2.96	0.6668	0.0			
Donor host family	Bovidae	3	1	27.86 [0–97.55]	2.47	0.8446	18.9	**0.0425∗**	0.00	0.00
	Canidae	7	6	92.94 [46.20–100]	3.17	0.8446	0.0			
	Rodentia	3	0	0 [0–61.92]	0.00	0.8446	0.0			
Receiver host family	Bovidae	2	1	50.0 [0–100]	1.85	0.4748	46.0	0.3582	0.00	0.00
	Canidae	1	1	100 [0–100]	0.00	0.4748	–			
	ND	4	3	82.18 [16.67–100]	2.78	0.4748	0.0			
	Rodentia	5	1	12.42 [0–70.74]	2.69	0.4748	0.0			
	Ticks	1	1	100 [0–100]	0.00	0.4748	–			
Parasite species	*L. chagasi (*Syn *L.infantum)*	2	1	50.0 [0–100]	1.85	0.5046	46.0	0.3655	0.00	0.00
	*L. infantum*	3	3	100 [38.08–100]	0.00	0.5046	0.0			
	*L. major*	1	0	0 [0–100]	0.00	0.5046	–			
	L. kala azar^*£*^	1	1	100 [0–100]	0.00	0.5046	–			
	*Leishmania* sp.	1	1	100 [0–100]	0.00	0.5046	–			
	*T. cruzi*	1	0	0 [0–100]	0.00	0.5046	–			
	*T. evansi*	1	0	0 [0–100]	0.00	0.5046	–			
	*T.theileri*	3	1	27.86 [0–97.55]	2.47	0.5046	18.9			

∗0.05; ∗∗0.01; ∗∗∗0.001 ^**£**^ L. kala azar refers to members of the *L. donovani* complex (*L. infantum* and *L. donovani*) without any other information on parasite typing at the time of the study. We therefore use the term proposed by the author. # ticks (receiver host) were infected via the inoculation of *A. americanum*-infected hemolymph (donor host).

For tick families, positivity rates were 100 % (95 % CI: 81.65–100) for Argasidae and 78.01 (95 % CI: 48.9–98.84 %) for Ixodidae, with no statistical difference according to tick family (p > 0.05). Slight variations were recorded for the genus, with no statistically significant difference. For the genera *Ornithodoros* and *Amblyomma*, all publications reported positive detection after the blood meal; for the genera *Rhipicephalus* and *Hyalomma*, detection rates were 74.57 % and 82.18 %, respectively. A large variation in prevalence is recorded at the species level, related to the low number of studies published (Table 4).

• *Parasite* genus

The highest detection rate was 99.11 % (95 % CI: 80.73–100) for *Trypanosoma*, whereas *Leishmania* was detected in 69.10 % (95 % CI: 62.64–97.17) of the studies. However, this difference is not statistically significant (P > 0.05) (Table 4).

• *Host* family

No statistical differences were recorded (Table 4).

#### 4.2.2 Infection by injection of tick-infected material

The meta-analysis includes 22 scientific papers. The common-effects model was used to analyze associated factors. About 72 % (95 % CI: 42–95 %) of articles show positive infection results. (Supplementary File Fig. S2).

• Tick's family, genus and species

The infection rate elicited via the injection of Argasidae-infected material was statistically higher than of Ixodidae-infected ones (rate of 85.25 % vs. 55.23 %). The highest rate, 85.25 %, was recorded for *Ornithodoros*, followed by *Rhipicephalus* and *Hyalomma*. However, if all these observations were not statistically significant, they interestingly point to some specificity according to the tick genus (Table 4).

• Parasite's genus and species

No significant difference was recorded (p > 0.05). Although we observed a generally higher infection rate with *T. cruzi*, *T. theileri, Leishmania*. sp., *L. chagasi* (Syn L. *infantum*), and *L. infantum* infected tick material.

• Host family

These analyses disclosed that tick material collected from infected Camelidae was more likely to initiate infection when injected into a noninfected recipient and Canidae appears to be more susceptible to infection when injected with infected tick material.

#### 4.2.3 Transmission through tick's blood feeding on a non-infected host

Tick-borne pathogen transmission can occur via mechanical or biological means. In mechanical transmission, ticks act as carriers, transferring pathogens between hosts without mandatory pathogen development within the tick. In contrast, biological transmission involves the pathogen undergoing necessary biological changes or replication within the tick, completing part of its life cycle before infecting the next host. We performed a meta-analysis on data extracted from 21 publications dealing with experimental transmission by ticks *via* blood feeding (Table 4). This analysis showed 34 % (95 % CI: 8–64 %) of studies confirming transmission (Supplementary Fig. S3). The common-effects model was used to analyze associated factors.

• Tick's family

The Ixodidae appeared to be more able to transmit Trypanosomatidae of medical or veterinary interest than Argasidae, with rates of 34.33 % (95 % CI: 0–94 %) and 23.7 % (95 % CI: 0–66.4 %), respectively (Table 4). Regarding the genus, minor variations were recorded without significant differences (p > 0.05). Regarding tick species, *R. sanguineus*, *O. lahorensis*, *O. crossi*, and *H. a. anatolicum* seemed more able to transmit Trypanosomatidae parasites of medical and veterinary interest. However, the sample size is too small to get insight into the statistical significance of these observations.

• Parasite genus

Although the success of transmission attempts is higher with *Leishmania* (82.18 % CI:35.16–100 %) than with *Trypanosoma* (12.42 % CI: 0–70.74 %), the meta-analysis does not record a statistically significant difference (p > 0.05) (Table 4).

• Donor and receiver host family

Surprisingly, the donor host appears to be a factor influencing the subsequent transmission of the pathogen during tick blood feeding (p < 0.05) Table 4. The infection rate for ticks varied from 100 % to 95 % (CI: 21.26–100 %) in Camelidae, to 59.68 % for Canidae and 50.0 % for Bovidae. The eight studies focusing on ticks collected on infected rodents reported no transmission. The host recipient also showed heterogeneity, with Canidae and Bovidae being more frequently infected at a rate of 72.14 % [2.45–100 %] than rodents (8.01 %, [0–41.38 %]).

#### 4.2.4 Vertical transmission

Our analysis includes only 13 studies focusing on the vertical transmission of Trypanosomatidae of medical or veterinary interest by ticks, with 55 % supporting the vertical transmission, as detailed in the supplementary file (Supplementary Fig. S4). The factors associated with this transmission were evaluated using the common-effect model. Despite the small sample size, vertical transmission is recorded at 61.27 % [22.41–94.58] for ticks of the Ixodidae family and 0 % for the Argasidae, although only one study deals with this issue. No significant difference exists according to genus and species (Table 4). Concerning genus and parasite species, parasites belonging to the *Leishmania* genus were better adapted to vertical transmission (82.18 [35.16–100]) than those of the *Trypanosoma* genus (12.42 [0–70.74]). Parasites from the *L. donovani* complex (*L. donovani* or *L. infantum*) appear to be adapted to vertical transmission. Also, the host family from which ticks feed plays a significant role in variability (p = 0.0425). Ticks feeding on dogs (Canidae) have the highest vertical transmission rate at 92.94 % [46.20–100 %], followed by those feeding on Bovidae at 27.86 % [0–97.55], and lastly, rodents with no vertical transmission of Trypanosomatidae in ticks following blood meal on rodent infected host.

## 5 Discussion

Biologists have shown particular interest in insects that serve as vectors for Trypanosomatidae due to the significant impact these parasites have on various animal species, including humans [147]. While nearly all Trypanosomatidae are transmitted by insects, a single publication documented an avian trypanosome transmitted by a non-traditional vector: an arachnid from the subclass Acari [153]. This anomaly spurred further investigations into the presence of Trypanosomatids within ticks [154]. Consequently, there has been an increased focus on understanding the potential role of ticks in harboring and transmitting these pathogenic protozoans.

To determine the prevalence of Trypanosomatidae of medical and veterinary interest in wild-caught ticks from endemic areas, both molecular (PCR, qPCR, PCR-HRM) and parasitological/immunological (microscopic examination, IHC) methods were employed. Molecular techniques that amplify genomic DNA, which can persist after parasite death, are more sensitive than parasitological/immunological methods that target living parasites or their immunological determinants, which degrade rapidly upon the death of the parasite. Statistical differences in detection rates were observed depending on the method used. Specifically, the highest prevalence (17.55 %) was recorded using molecular methods, compared to only 0.1 % for microscopic detection of parasites. The presence of DNA or parasites in the digestive systems of blood-fed ticks collected from hosts has limited predictive value for inferring a vectorial role. Most publications included in our systematic review and meta-analysis focused on ticks collected directly from hosts, concentrating on detecting pathogens from host blood deposited in tick bodies. DNA detection alone is insufficient, particularly in the case of ticks, due to their digestive capabilities and other metabolic peculiarities. To further analyze the prevalence of Trypanosomatidae in field-collected tick samples, it will be necessary to isolate the parasites themselves from these ticks and/or detect parasite-specific mRNA, which serves as a better indicator of parasite survival in the digestive tract and other tick organs.

Data on field tick infections reveal that the detection of Trypanosomatidae of medical or veterinary interest in ticks varies significantly, ranging from 0.01 % to 1.26 % in some regions to as high as 14.27 %–37.83 % in others, depending on the country or continent (Table 3). Many studies utilize pooled samples to ascertain prevalence, which can greatly overestimate the results. Nonetheless, the meta-analysis provides initial evidence of a high frequency of contact between infected hosts and ticks, a crucial factor for facilitating transmission by arthropod vectors. Unfortunately, in many regions where Trypanosomatidae infections are common, there is a lack of available information regarding the infection rates of these pathogenic agents in their proven vectors and/or in ticks. For instance, while the Mediterranean area has a high incidence of leishmaniases [155], there is no data on the carriage of *Leishmania* by ticks in countries such as Egypt, Libya, and Morocco. Similarly, Latin America and the Caribbean are affected by Chagas disease, caused by *T. cruzi* [156], yet no information on its presence in ticks is available. This lack of data complicates efforts to conduct a global analysis of the seasonal activity patterns of ticks in relation to the incidence of Trypanosomatidae pathogen infections in hosts, as well as the geographical overlap between tick populations and human or animal infections caused by Trypanosomatidae pathogens.

If 996 species of ticks (774 hard ticks and 221 soft ticks) are described worldwide [3,4], with 25 species acting as vectors of major diseases, the presence of Trypanosomatidae pathogens of medical or veterinary interest has been investigated in twenty tick species. In South America, 137 species of hard ticks from five genera and 87 species of soft ticks have been reported [157,158]. All studies collected for this review focused on the Ixodidae family, with no field data available on soft ticks from the Argasidae family. The detection rate among members of the *Rhipicephalus* genus is the highest at 17.49 %, followed by *Amblyomma* (11.47 %), *Hyalomma* (2.68 %), and *Ixodes* (1.87 %), while *Dermacentor* and *Haemaphysalis* ticks were negative.

Variability in field studies regarding the detection of Trypanosomatidae in ticks can often be attributed to host effects. Specifically, ticks harvested from Canidae—predominantly non-questing ticks belonging to the *Rhipicephalus* genus—exhibit a higher detection rate of pathogens compared to ticks collected from other mammals (P = 0.0007). This discrepancy may partly result from the close relationship between dogs and humans, coupled with dogs' heightened susceptibility to *T. cruzi*, which could increase the risk of tick infection [159]. Additionally, dogs serve as significant reservoirs for several *Leishmania* species, including *L. infantum*, *L. peruviana*, and *L. donovani* [160].

The extensive geographical distribution of ticks belonging to the genus *Rhipicephalus*, their wide range of animal hosts, known vector competence, and diverse morphology provide strong arguments for this meta-analysis on their potential role in the transmission of Trypanosomatidae [161]. *Rhipicephalus* *sanguineus* is a representative species of this genus, particularly due to its close association with domestic dogs, which are known reservoirs for *Leishmania*. This connection highlights the likely involvement of this tick species in the persistence and transmission of these parasites in natural habitats [162].

The concept of vector competence refers to the innate ability of an arthropod to harbor and transmit microbial agents [163,164]. Establishing the vector capacity of a tick involves confirming its ability to become infected during a blood meal on a host, facilitating the multiplication of the pathogen prior to transmission through saliva, and maintaining the pathogen throughout the tick's developmental stages for potential vertical transmission [165].

Regarding parasite acquisition, the meta-analysis reveals an efficient tick infection following blood feeding in experimental settings, with 31 successful infections out of 37 attempts (Table 4). While no significant factors were associated with parasite acquisition from an infected host (donor host), the analysis does indicate some trends worth discussing. Firstly, Argasidae ticks appear more likely to become infected compared to Ixodidae ticks when feeding on an infected host (P = 0.0976). The nature of the host does not seem to influence tick infection rates; however, the family of the parasite does impact infection rates. Specifically, ticks have a higher infection rate when feeding on a *Trypanosoma*-infected host compared to a *Leishmania*-infected host, with tick infection being more efficient when they take a blood meal from a host infected with *Trypanosoma* (P = 0.0794) (Table 4).

*Trypanosoma cruzi* and *T. evansi* are particularly likely to infect ticks following blood feeding. These observations may relate to the intrinsic ability of trypanosomes to multiply in the blood of the infected host, increasing their availability to ticks during the blood-feeding process. Additionally, the disparity in infection rates might be attributed to differing feeding behaviors between the two tick families: Ixodidae ticks attach to the host's skin and feed slowly over several days, whereas Argasidae ticks rapidly ingest large volumes of blood in a short time (20–70 min), facilitating a more efficient uptake of infectious agents, especially under experimental conditions [166].

The rapid feeding behavior of Argasidae ticks enhances their efficiency at acquiring blood-circulating parasites like *T. brucei* or *T. evansi*, compared to the slower-feeding Ixodidae ticks, which may be less effective at acquiring tissue-located parasites. Furthermore, the prolonged feeding process of Ixodidae ticks, lasting up to two weeks, exposes engorged pathogens to the full spectrum of host defense mechanisms, including specific acquired immunity that may impact the survival of Trypanosomatidae [167].

Information gathered on the efficiency of infection through the injection of contaminated tick material provides insight into the presence of infectious parasitic stages in infected ticks. Our meta-analysis did not identify any significant factors related to the donor or receiver host, ticks, or parasites. While Argasidae ticks appear more efficient in such transmission than Ixodidae (7 successes out of 9 attempts compared to 7 successes out of 13 attempts), these differences are not statistically significant (P = 0.2884).

Since ticks feed only once at each life stage, vertical transmission of pathogens is a crucial factor to consider when addressing the vectorial status of ticks for Trypanosomatidae parasites. The literature survey indicates that vertical transmission occurs in experimental settings, and only in Ixodidae ticks. Although not statistically significant, *Leishmania* seems more likely to be vertically transmitted in R. sanguineus than *Trypanosoma* species (P = 0.0634). Interestingly, *Babesia* can undergo transovarial transmission in ticks, suggesting potential similar behavior for Trypanosomatidae [168]. However, the limited number of studies on vertical transmission restricts further discussion on the influence of tick identity or parasite species on vertical transmission.

*Trypanosoma evansi* and *T. vivax* are mechanically transmitted pathogens, meaning they are spread from host to host without undergoing biological replication within their vector. This mode of transmission does not align well with the feeding habits of ticks, except in instances of interrupted feeding [169]. While there is limited data on *T. vivax*, a relatively good success rate of 3 out of 6 attempts was recorded for the transmission of *T. evansi* via tick blood feeding. Therefore, the transmission of *T. evansi* or *T. vivax* following interrupted blood feeding warrants further investigation.

Salivarian trypanosomes, such as *T. brucei*, *T. evansi*, *T. congolense*, *T. vivax*, and *Leishmania* parasites, are transmitted through blood-feeding via the injection or regurgitation of saliva during the feeding process. In contrast, stercorarian trypanosomes, like *T. cruzi*, infect their hosts through the deposition of the pathogen on the host's skin during blood-feeding, subsequently entering the host through scratching of the infected blood onto mucosal surfaces. Infection via ingestion is also documented for *T. cruzi* [170].

Considering this aspect, we observed more successful attempts to transmit Trypanosomatidae pathogens through the blood-feeding of infected ticks harboring salivarian trypanosomes, such as *T. evansi*, T*. lewisi*, and *T. theileri*, compared to those with stercorarian pathogens. Specifically, there were 11 attempts with 5 successes for salivarian trypanosomes, and 6 attempts for *T. cruzi*. The data indicate a general trend of higher infection rates for salivarian Trypanosomatidae during tick blood meals. However, these differences are not statistically significant, preventing us from drawing definitive conclusions about the impact of the transmission route.

Overall, this systematic review and meta-analysis provide an updated overview of the vector status of ticks for Trypanosomatidae. This perspective highlights the limited information available regarding the presence of Trypanosomatidae infecting humans and animals of veterinary importance in field-collected specimens or during experimental studies. Specifically, data are available for 20 species out of the 774 recognized hard ticks and 221 soft ticks. Of the 23 *Leishmania* and 11 *Trypanosoma* species of medical or veterinary interest, we gathered information on 6 species from the *Leishmania* genus and 9 from the *Trypanosoma* genus. Notably, we also collected data on the presence of trypanosomes associated with human African trypanosomiasis (*T. b. gambiense* and *T. b. rhodesiense*) in field-collected ticks.

However, we could not provide conclusive quantitative evidence regarding the vectorial role of ticks for *Leishmania* and *Trypanosoma* parasites in medical or veterinary contexts. For R. sanguineus, the documentation of Trypanosomatidae infecting humans and animals is the most extensive, with 21 studies. The capacity of this tick species to acquire parasites during blood feeding on infected hosts has been successfully demonstrated in 11 out of 15 experiments. Additionally, the presence of the infective parasite stage has been assessed through the injection of tick-infected material (7 attempts with 5 successes), as well as re-transmission via infected tick blood feeding (6 attempts with 3 successes). Importantly, data on vertical transmission have also been collected (9 attempts with 7 successes).

While most of these collected data pertain to *L. infantum*, they collectively support the vectorial competence of *R. sanguineus*, which now needs to be more thoroughly demonstrated using advanced molecular methods in field-collected specimens alongside additional experimental evidence. Furthermore, the presence of Trypanosomatidae parasites with medical or veterinary significance in ticks is not uncommon, and ticks from the Argasidae family may also play a role in the transmission of these pathogens. The precise role of ticks in sustaining both the parasitic developmental and epidemiological cycles of Trypanosomatidae requires further investigation and continuous scrutiny.

## CRediT authorship contribution statement

**Tahar Kernif:** Writing – review & editing, Writing – original draft, Visualization, Validation, Methodology, Investigation, Funding acquisition, Formal analysis, Data curation, Conceptualization. **Bachir Medrouh:** Writing – review & editing, Formal analysis, Data curation. **Naouel Eddaikra:** Writing – review & editing, Data curation. **Bruno Oury:** Writing – review & editing, Writing – original draft, Formal analysis. **Philippe Holzmuller:** Writing – review & editing, Writing – original draft, Formal analysis, Data curation, Conceptualization. **Denis Sereno:** Writing – review & editing, Writing – original draft, Visualization, Validation, Supervision, Resources, Project administration, Methodology, Investigation, Funding acquisition, Formal analysis, Data curation, Conceptualization.

## Informed consent statement

Not applicable.

## Institutional review board statement

The study was conducted according to the guidelines of the Preferred Reporting Items for Systematic Reviews and Meta-Analyses (PRISMA).

## Data availability statement

Data is contained within the article and Supplementary Material.

## Declaration of generative AI and AI-assisted technologies in the writing process

During the preparation of this work the authors used ChatGPT 4.0 in order to correct grammar and spelling. After using this tool/service, the authors reviewed and edited the content as needed and take full responsibility for the content of the published article.

## Funding

This research was funded by LeiSHeild-RISE MATI project, Grant Agreement N°778298.

## Declaration of competing interest

The authors declare the following financial interests/personal relationships which may be considered as potential competing interests:Kernif Tahar reports financial support and travel were provided by 10.13039/501100000780European Union. Sereno Denis reports financial support, administrative support, and article publishing charges were provided by 10.13039/501100000780European Union. Denis Sereno reports a relationship with 10.13039/501100000780European Union that includes: funding grants. Kernif Tahar reports a relationship with 10.13039/501100000780European Union that includes: funding grants and travel reimbursement. No additional information If there are other authors, they declare that they have no known competing financial interests or personal relationships that could have appeared to influence the work reported in this paper.
