# Supplementary material for: Ticks as vectors of Trypanosomatidae with medical or veterinary interest: Insights and implications from a comprehensive systematic review and meta-analysis
Source: Heliyon. 2024 Dec 5;10(24):e40895. doi: 10.1016/j.heliyon.2024.e40895 (PMC11698932; doi:10.1016/j.heliyon.2024.e40895)
Supplement: Multimedia component 1 [file mmc1.docx]

Supplementary material, Data collection sheet.

| **Author (s)** | **Publication Year** | **Continent** | **Country** | **City** | **Family** | **Species** | **Tick's developmental stages** | **Tick Identification method (by key (K) or molecular (M)=** | **Host (Family)** | **Host (Species)** | **Positive (Y/N)** | **Number of ticks examined** | **N positive ticks** | **Leish (L) or Tryp (T) or both** | **Localization in the tick (F:faeces; G: gut; Gr: crushing; H:haemolymph; O: ovaries; SG: salivary glands )** | **Trypanoaomatidae species** | **Positive %** | **Methodology of Trypansomatidae identification in ticks** | **Methodology of Trypanosomatidae identification in host** |
| --- | --- | --- | --- | --- | --- | --- | --- | --- | --- | --- | --- | --- | --- | --- | --- | --- | --- | --- | --- |
| P Giraud | 1934 | Europe | France | Marseille | Ixodidae | *Rhipicephalus sanguineus* | NS | NS | Hominidae | *Homo sapiens* | Y | NS | NS | L | NA | *Leishmania_*Kala Azar | NS | NS | NS |
| F Pifano | 1941 | South America | Venezuela | Yaracuy | Ixodidae | *Amblyomma longirostrum* | Larvae | K | Cercolabidae | *Arboreal porcupine* | Y | NS | NS | T | NA | *Trypanosoma cruzi* | NS | NS | NS |
| F VACHON, G RENAULT, JP MARCHETTI | 1983 | Africa | Gabon | Port Gentil | NS | NS | NS | NS | Hominidae | *Homo sapiens* | N | NS | NS | T | NA | *Trypanosoma spp.* | NS | NS | Serology |
| Aeschlimann A, Burgdorfer W, Matile H, Peter O, Wyler R. | 1979 | Europe | Switzerland |  | Ixodidae | *Ixodes ricinus* | Nymph and Adult | K | None |  | Y | 2501 | 5 | T | Hemolymph | *Trypanosoma theileri* | 0.19 | Microscopy | NS |
| Yousfi Monod R, Aeschlimann A, Derscheid JM. | 1986 | Africa | Algeria | Oran | Ixodidae | *Hyalomma detritum* | Nymph and Adult | K | Bovidae; Canidae | *Bos taurus; Canis lupus* | Y | 1 | 1 | T | Hemolymph | *Trypanosoma theileri* | 100 | Microscopy | NS |
| Yousfi Monod R, Aeschlimann A, Derscheid JM. | 1986 | Africa | Algeria | Oran | Ixodidae | *Hyalomma marginatum* | Nymph and Adult | K | Bovidae; Canidae | *Bos taurus; Canis lupus* | N | 2 | 0 | T | Hemolymph | *Trypanosoma theileri ; Trypanosoma evansi* | 0 | Microscopy | NS |
| Yousfi Monod R, Aeschlimann A, Derscheid JM. | 1986 | Africa | Algeria | Oran | Ixodidae | *Hyalomma lusitanicum* | Nymph and Adult | K | Bovidae; Canidae | *Bos taurus; Canis lupus* | N | 3 | 0 | T | Hemolymph | *Trypanosoma theileri; Trypanosoma evansi* | 0 | Microscopy | NS |
| Yousfi Monod R, Aeschlimann A, Derscheid JM. | 1986 | Africa | Algeria | Oran | Ixodidae | *Hyalomma excavatum* | Nymph and Adult | K | Bovidae; Canidae | *Bos taurus; Canis lupus* | N | 2 | 0 | T | Hemolymph | *Trypanosoma theileri; Trypanosoma evansi* | 0 | Microscopy | NS |
| Yousfi Monod R, Aeschlimann A, Derscheid JM. | 1986 | Africa | Algeria | Oran | Ixodidae | *Rhipicephalus sanguineus* | Nymph and Adult | K | Bovidae; Canidae | *Bos taurus; Canis lupus* | Y | 250 | 5 | T | Hemolymph | *Trypanosoma evansi* | 2 | Microscopy | NS |
| Yousfi Monod R, Aeschlimann A, Derscheid JM. | 1986 | Africa | Algeria | Oran | Ixodidae | *Rhipicephalus turanicus* | Nymph and Adult | K | Bovidae; Canidae | *Bos taurus; Canis lupus* | N | 23 | 0 | T | Hemolymph | *Trypanosoma theileri; Trypanosoma evansi* | 0 | Microscopy | NS |
| Yousfi Monod R, Aeschlimann A, Derscheid JM. | 1986 | Europe | Switzerland | Plateau bernois | Ixodidae | *Ixodes ricinus* | Nymph and Adult | K | Bovidae; Canidae | *Bos taurus; Canis lupus* | Y | 3688 | 8 | T | Hemolymph | *Trypanosoma theileri* | 0.2 | Microscopy | NS |
| F Bacellar, MS Núncio, AR Filipe | 1990 | Europe | Portugal |  | Ixodidae | *Hyalomma marginatum* | Adult | K | Bovidae | *Bos taurus* | Y | NS | NS | T | Hemolymph | *Trypanosoma theileri* | NS | Microscopy | NS |
| СР Постоян | 2001 | South America | Cuba | Oriente | Ixodidae | *Rhipicephalus (Boophilus) microplus* | Adult | K | Bovidae | *Bos taurus* | Y | 28 | NS | T | Gut | *Trypanosoma vivax* | NS | Microscopy | NS |
| СР Постоян | 2001 | South America | Cuba | Oriente | Ixodidae | *Amblyomma cajennense* | Adult | K | Bovidae | *Bos taurus* | Y | 35 | NS | T | Gut | *Trypanosoma vivax* | NS | Microscopy | NS |
| СР Постоян | 2001 | South America | Cuba | Oriente | Ixodidae | *Rhipicephalus (Boophilus) microplus* | Adult | K | Bovidae | *Bos taurus* | N | 28 | NS | T | Hemolymph | *Trypanosoma vivax* | NS | Microscopy | NS |
| СР Постоян | 2001 | South America | Cuba | Oriente | Ixodidae | *Amblyomma cajennense* | Adult | K | Bovidae | *Bos taurus* | N | 35 | NS | T | Hemolymph | *Trypanosoma vivax* | NS | Microscopy | NS |
| Martins JR, Leite RC, Doyle RL. | 2008 | South America | Brazil | Rio Grande do Sul | Ixodidae | *Rhipicephalus (Boophilus) microplus* | NA | K | Bovidae | *Bos sp.* | Y | NS | NS | T | Hemolymph | *Trypanosoma theileri-like* | NS | ND | Antibody Test, or IFAT |
| FD Torres | 2009 | South America | Brazil | Pernambuco | Ixodidae | *Rhipicephalus sanguineus* | NA | K | Canidae | *Canis lupus* | Y | 94 | 13 | L | Crushed ticks | *Leishmania infantum* | 13.8 | PCR; qPCR | IFAT, PCR, real time qPCR |
| Dantas-Torres F, de Paiva-Cavalcanti M, Figueredo LA, Melo MF, da Silva FJ, da Silva AL, Almeida EL, Brandão-Filho SP. | 2010 | South America | Brazil | Pernambuco | Ixodidae | *Amblyomma ovale* | Nymph and Adult | K | Canidae | *Canis lupus* | N | 10 | 0 | L | Crushed ticks | *Leishmania infantum* | 0 | qPCR | IFAT |
| Dantas-Torres F, de Paiva-Cavalcanti M, Figueredo LA, Melo MF, da Silva FJ, da Silva AL, Almeida EL, Brandão-Filho SP. | 2010 | South America | Brazil | Pernambuco | Ixodidae | *Rhipicephalus sanguineus* | Nymph and Adult | K | Canidae | *Canis lupus* | N | 10 | 0 | L | Crushed ticks | *Leishmania infantum* | 0 | qPCR | IFAT |
| Dantas-Torres F, Lorusso V, Testini G, de Paiva-Cavalcanti M, Figueredo LA, Stanneck D, Mencke N, Brandão-Filho SP, Alves LC, Otranto D. | 2010 | Europe | Italy | Basilicata | Ixodidae | *Rhipicephalus sanguineus* | Adult | K | Canidae | *Canis lupus* | Y | 4 pools (22) | 2 | L | Crushed ticks, Saliary Gland | *Leishmania infantum* | 50 | PCR; qPCR; sequencing | NS |
| Dantas-Torres F, Lorusso V, Testini G, de Paiva-Cavalcanti M, Figueredo LA, Stanneck D, Mencke N, Brandão-Filho SP, Alves LC, Otranto D. | 2010 | South America | Brazil | Pernambuco | Ixodidae | *Rhipicephalus sanguineus* | Adult | K | Canidae | *Canis lupus* | Y | 73 | 9 | L | Crushed ticks, Saliary Gland | *Leishmania infantum* | 12.3 | PCR; qPCR; sequencing | NS |
| Paz GF, Ribeiro MF, de Magalhães DF, Sathler KP, Morais MH, Fiúza VO, Brandão ST, Werneck GL, Fortes-Dias CL, Dias ES. | 2010 | South America | Brazil | Minas Gerais | Ixodidae | *Rhipicephalus sanguineus* | NA | NS | Canidae | *Canis lupus* | N | NS | NS | L | NA | *Leishmania* sp. | NS | present of tick or no | IFAT |
| OA da Silva, GMS Braga | 2010 | South America | Brazil | Pernambuco | Ixodidae | *Rhipicephalus sanguineus* | NA | K | Canidae | *Canis lupus* | Y | NS | NS | L | Gut | *Leishmania* sp. | NS | Microscopy | IFAT |
| RMFN Odorizzi | 2010 | South America | Brazil | São Paulo | Ixodidae | *Rhipicephalus sanguineus* | Larva, Nymph and Adult | K | Canidae | *Canis lupus* | Y | 208 | 105 | L | Crushed ticks | *Leishmania infantum* | 50.5 | PCR | ELISA |
| Satta G, Chisu V, Cabras P, Fois F, Masala G. | 2011 | Europe | Italy | Sardinia | Ixodidae | *Rhipicephalus sanguineus* | Adult | K | Canidae | *Canis familiaris* | N | 209 pools | 0 | L | Crushed ticks | *Leishmania infantum* | 0 | PCR | NS |
| Satta G, Chisu V, Cabras P, Fois F, Masala G. | 2011 | Europe | Italy | Sardinia | Ixodidae | *Rhipicephalus turanicus* | Adult | K | Bovidae | *Ovis spp. Capara spp.* | N | 58 | 0 | L | Crushed ticks | *Leishmania infantum* | 0 | PCR | NS |
| Satta G, Chisu V, Cabras P, Fois F, Masala G. | 2011 | Europe | Italy | Sardinia | Ixodidae | *Rhipicephalus bursa* | Adult | K | Bovidae, cervidae, Equidae | *Ovis spp. Capra spp. Bos spp, Equus spp. Cervus spp..* | N | 18 | 0 | L | Crushed ticks | *Leishmania infantum* | 0 | PCR | NS |
| Satta G, Chisu V, Cabras P, Fois F, Masala G. | 2011 | Europe | Italy | Sardinia | Ixodidae | *Rhipicephalus pusillus* | Adult | K | Hedgehogs |  | N | 1 | 0 | L | Crushed ticks | *Leishmania infantum* | 0 | PCR | NS |
| Satta G, Chisu V, Cabras P, Fois F, Masala G. | 2011 | Europe | Italy | Sardinia | Ixodidae | *Hyalomma marginatum* | Adult | K | Cattle |  | N | 3 | 0 | L | Crushed ticks | *Leishmania infantum* | 0 | PCR | NS |
| Satta G, Chisu V, Cabras P, Fois F, Masala G. | 2011 | Europe | Italy | Sardinia | Ixodidae | *Haemaphysalis sulcata* | Adult | K | Bovidae | *Ovis spp. Capara spp.* | N | 1 | 0 | L | Crushed ticks | *Leishmania infantum* | 0 | PCR | NS |
| Satta G, Chisu V, Cabras P, Fois F, Masala G. | 2011 | Europe | Italy | Sardinia | Ixodidae | *Dermacentor marginatus* | Adult | K | Wild boars |  | N | 3 | 0 | L | Crushed ticks | *Leishmania infantum* | 0 | PCR | NS |
| Colombo FA, Odorizzi RM, Laurenti MD, Galati EA, Canavez F, Pereira-Chioccola VL. | 2011 | South America | Brazil | São Paulo | Ixodidae | *Rhipicephalus sanguineus* | Nymph and Adult | NS | Canidae | *Canis lupus* | Y | 91 | 64 | L | Crushed ticks | *Leishmania infantum* | 70.3 | PCR, reverse transcriptase RT-PCR, Sequencing | ELISA, PCR |
| Solano-Gallego L, Rossi L, Scroccaro AM, Montarsi F, Caldin M, Furlanello T, Trotta M. | 2012 | Europe | Italy | Toscane; Venice | Ixodidae | *Rhipicephalus sanguineus* | Adult | K | Canidae | *Canis lupus* | Y | 128 | 13 | L | Crushed ticks | *Leishmania infantum* | 10.1 | qPCR | ELISA, PCR |
| Trotta M, Nicetto M, Fogliazza A, Montarsi F, Caldin M, Furlanello T, Solano-Gallego L. | 2012 | Europe | Italy | Throughout (South, Central, North) Italy | Ixodidae | *Rhipicephalus sanguineus* | Nymph and Adult | K | Canidae | *Canis lupus* | Y | 78 | 2 | L | Crushed ticks | *Leishmania infantum* | 2.5 | qPCR | ELISA, PCR |
| Trotta M, Nicetto M, Fogliazza A, Montarsi F, Caldin M, Furlanello T, Solano-Gallego L. | 2012 | Europe | Italy | Throughout (South, Central, North) Italy | Ixodidae | *Dermacentor marginatus* | Nymph and Adult | K | Canidae | *Canis lupus* | N | 3 | 0 | L | Crushed ticks | *Leishmania infantum* | 0 | qPCR | ELISA, PCR |
| Trotta M, Nicetto M, Fogliazza A, Montarsi F, Caldin M, Furlanello T, Solano-Gallego L. | 2012 | Europe | Italy | Throughout (South, Central, North) Italy | Ixodidae | *Ixode ricinus* | Nymph and Adult | K | Canidae | *Canis lupus* | Y | 10 | 5 | L | Crushed ticks | *Leishmania infantum* | 50 | qPCR | ELISA, PCR |
| de Morais RC, Gonçalves Sda C, Costa PL, da Silva KG, da Silva FJ, Silva RP, de Brito ME, Brandão-Filho SP, Dantas-Torres F, de Paiva-Cavalcanti M. | 2013 | South America | Brazil | Pernambuco | Ixodidae | *Rhipicephalus sanguineus* | Adult | K | Canidae | *Canis lupus* | Y | 75 | 30 | L | Crushed ticks | *Leishmania infantum* | 40.0 | PCR, qPCR, seuqencing | PCR, qPCR |
| de Morais RC, Gonçalves-de-Albuquerque Sda C, Pessoa e Silva R, Costa PL, da Silva KG, da Silva FJ, Brandão-Filho SP, Dantas-Torres F, de Paiva-Cavalcanti M. | 2013 | South America | Brazil | Pernambuco | Ixodidae | *Rhipicephalus sanguineus* | Adult | K | Canidae | *Canis lupus* | Y | 75 | 32 | L | Crushed ticks | *Leishmania braziliensis* | 42.6 | PCR, qPCR | NS |
| Paz GF, Reis IA, Avelar DM, da Mata Ferreira EC, Werneck GL. | 2013 | South America | Brazil | Minas Gerais | Ixodidae | *Rhipicephalus sanguineus* | NA | NS | Canidae | *Canis lupus* | N | NS | NS | L | NA | *Leishmania infantum* | NS | infestation | IFAT, ELISA |
| AM Bolivar | 2013 | South America | Venezuela | Mérida | Ixodidae | *Rhipicephalus (Boophilus) microplus* | NA | K | Bovidae | *Bos taurus* | Y | 285 | 22 | T | Crushed ticks | *Trypanosoma vivax* | 7.7 | PCR | NS |
| de Almeida, Robson Ferreira Cavalcante; Garcia, Marcos Valério; Cunha, Rodrigo Casquero; Matias, Jaqueline; e Silva, Elaine Araújo; de Fatima Cepa Matos, Maria; Andreotti, Renato; | 2013 | South America | Brazil | Campo Grande | Ixodidae | *Rhipicephalus sanguineus* | Adult | K | Canidae | *Canis lupus* | Y | 420 | 107 | L | Hemolymph, Crushed ticks | *Leishmania chagasi* | 25.5 | Microscopy, PCR | NS |
| de Almeida, Robson Ferreira Cavalcante; Garcia, Marcos Valério; Cunha, Rodrigo Casquero; Matias, Jaqueline; e Silva, Elaine Araújo; de Fatima Cepa Matos, Maria; Andreotti, Renato; | 2013 | South America | Brazil | Campo Grande | Ixodidae | *Amblyomma cajennense* | Adult | K | Canidae | *Canis lupus* | N | 24 | 0 | L | Hemolymph, Crushed ticks | *Leishmania chagasi* | 0 | Microscopy, PCR | NS |
| de Almeida, Robson Ferreira Cavalcante; Garcia, Marcos Valério; Cunha, Rodrigo Casquero; Matias, Jaqueline; e Silva, Elaine Araújo; de Fatima Cepa Matos, Maria; Andreotti, Renato; | 2013 | South America | Brazil | Campo Grande | Ixodidae | *Rhipicephalus sanguineus* | Adult | K | Canidae | *Canis lupus* | N | 420 | 0 | T | Hemolymph, Crushed ticks | *Trypanosoma cruzi* | 0 | Microscopy, PCR | NS |
| de Almeida, Robson Ferreira Cavalcante; Garcia, Marcos Valério; Cunha, Rodrigo Casquero; Matias, Jaqueline; e Silva, Elaine Araújo; de Fatima Cepa Matos, Maria; Andreotti, Renato; | 2013 | South America | Brazil | Campo Grande | Ixodidae | *Amblyomma cajennense* | Adult | K | Canidae | *Canis lupus* | N | 24 | 0 | T | Hemolymph, Crushed ticks | *Trypanosoma cruzi* | 0 | Microscopy, PCR | NS |
| Cominetti M. C., de Almeida R. F. C., Csordas B. G., Andreotti, R. | 2013 | South America | Brazil | Mato Grosso do Sul | Ixodidae | *Rhipicephalus sanguineus* | NA | K | Canidae | *Canis lupus* | Y | 63 | 7 | T | Crushed ticks | *Trypanosoma evansi;* | 11.1 | PCR | NS |
| Cominetti M. C., de Almeida R. F. C., Csordas B. G., Andreotti, R. | 2013 | South America | Brazil | Mato Grosso do Sul | Ixodidae | *Rhipicephalus sanguineus* | NA | K | Canidae | *Canis lupus* | Y | 63 | 15 | T | Crushed ticks | *Trypanosoma vivax* | 23.8 | PCR | NS |
| Chen Z, Liu Q, Liu JQ, Xu BL, Lv S, Xia S, Zhou XN. | 2014 | Asia | China | Xinyang | Ixodidae | *Haemaphysalis longicornis* | Larva , Nymph and Adult | K | sheep, cattle and dogs |  | N | 298 | 0 | L | Crushed ticks | *Leishmania infantum* | 0 | PCR | NS |
| Chen Z, Liu Q, Liu JQ, Xu BL, Lv S, Xia S, Zhou XN. | 2014 | Asia | China | Xinyang | Ixodidae | *Rhipicephalus (Boophilus) microplus* | Larva , Nymph and Adult | K | sheep, cattle and dogs |  | N | 10 | 0 | L | Crushed ticks | *Leishmania infantum* | 0 | PCR | NS |
| Campos JH, Costa FA. | 2014 | South America | Brazil | Piauí | Ixodidae | *Rhipicephalus sanguineus* | Adult | K | Canidae | *Canis lupus* | Y | 35 pools | 8 | L | Crushed ticks | *Leishmania infantum* | 22.8 | PCR | IFAT, ELISA |
| Gonçalves LR, Filgueira KD, Ahid SM, Pereira JS, Vale AM, Machado RZ, André MR. | 2014 | South America | Brazil | Rio Grande do Norte | Ixodidae | *Rhipicephalus sanguineus* | Adult | K | Canidae | *Canis lupus* | Y | 7 | 2 | L | Crushed ticks | *Leishmania infantum* | 28.6 | PCR, Sequencing | PCR, Sequencing |
| Salvatore D, Aureli S, Baldelli R, Di Francesco A, Tampieri MP, Galuppi R. | 2014 | Europe | Italy |  | Ixodidae | *Ixodes ricinus* | Adult | Key and Molecular | Dogs, horses, cat, bovine, humans |  | Y | 119 | 9 | L | Crushed ticks | *Leishmania infantum* | 7.5 | PCR | NS |
| Xu D, Zhang J, Shi Z, Song C, Zheng X, Zhang Y, Hao Y, Dong H, Wei L, El-Mahallawy HS, Kelly P, Xiong W, Wang H, Li J, Zhang X, Gu J, Wang C. | 2015 | Asia | China | Taixing | Ixodidae | *Rhipicephalus sanguineus* | NA | K | Canidae | *Canis lupus* | N | 146 | 0 | L | Crushed ticks | *Leishmania spp.* | 0 | qPCR | qPCR |
| Pennisi MG, Persichetti MF, Serrano L, Altet L, Reale S, Gulotta L, Solano-Gallego L. | 2015 | Europe | Italy | Sicily; Calabria | Ixodidae | *Rhipicephalus sanguineus* | Adult | Key | Felidae | *Felis catus* | Y | 28 | 3 | L | Crushed ticks | *Leishmania infantum* | 10.7 | PCR | NS |
| Pennisi MG, Persichetti MF, Serrano L, Altet L, Reale S, Gulotta L, Solano-Gallego L. | 2015 | Europe | Italy | Sicily; Calabria | Ixodidae | *Rhipicephalus pusillus* | Adult | Key | Felidae | *Felis catus* | Y | 17 | 3 | L | Crushed ticks | *Leishmania infantum* | 17.6 | PCR | NS |
| Pennisi MG, Persichetti MF, Serrano L, Altet L, Reale S, Gulotta L, Solano-Gallego L. | 2015 | Europe | Italy | Sicily; Calabria | Ixodidae | *Ixodes ricinus* | Adult | Key | Felidae | *Felis catus* | Y | 20 | 1 | L | Crushed ticks | *Leishmania infantum* | 5 | PCR | NS |
| Pennisi MG, Persichetti MF, Serrano L, Altet L, Reale S, Gulotta L, Solano-Gallego L. | 2015 | Europe | Italy | Sicily; Calabria | Ixodidae | *Ixodes ventalloi* | Adult | Key | Felidae | *Felis catus* | Y | 62 | 4 | L | Crushed ticks | *Leishmania infantum* | 6.25 | PCR | NS |
| Pennisi MG, Persichetti MF, Serrano L, Altet L, Reale S, Gulotta L, Solano-Gallego L. | 2015 | Europe | Italy | Sicily; Calabria | Ixodidae | *Ixodes spp.* | Adult | Key | Felidae | *Felis catus* | N | 5 | 0 | L | Crushed ticks | *Leishmania infantum* | 0 | PCR | NS |
| Medeiros-Silva V, Gurgel-Gonçalves R, Nitz N, Morales LE, Cruz LM, Sobral IG, Boité MC, Ferreira GE, Cupolillo E, Romero GA. | 2015 | South America | Brazil | Brasilia | Ixodidae | *Rhipicephalus sanguineus* | Nymph & Adult | Key | Canidae | *Canis lupus* | Y | 44 pools (Salivary glands) | 13 | L | Salivary Gland | *Leishmania infantum* | 29.5 | PCR, RFLP, seuqencing, Parasit culture | Rapid immunochromatographic test DPP, IFAT, ELISA, PCR |
| Medeiros-Silva V, Gurgel-Gonçalves R, Nitz N, Morales LE, Cruz LM, Sobral IG, Boité MC, Ferreira GE, Cupolillo E, Romero GA. | 2015 | South America | Brazil | Brasilia | Ixodidae | *Rhipicephalus sanguineus* | Nymph and Adult | Key | Canidae | *Canis lupus* | Y | 44 pools (Intestines) | 12 | L | Salivary Gland | *Leishmania infantum* | 27.3 | PCR, RFLP, seuqencing, Parasit culture | Rapid immunochromatographic test DPP, IFAT, ELISA, PCR |
| MA Viol | 2015 | South America | Brazil | Mato Grosso do Sul; São Paulo | Ixodidae | *Rhipicephalus sanguineus* | Adult | Key | Canidae | *Canis lupus* | Y | 99 pools | 93 | L | Gut | *Leishmania* sp. | 93.9 | qPCR | NS |
| MA Viol | 2015 | South America | Brazil | Mato Grosso do Sul; São Paulo | Ixodidae | *Rhipicephalus sanguineus* | Adult | Key | Canidae | *Canis lupus* | Y | 99 pools | 47 | L | Ovary | *Leishmania* sp. | 46.9 | qPCR | NS |
| MA Viol | 2015 | South America | Brazil | Mato Grosso do Sul; São Paulo | Ixodidae | *Rhipicephalus sanguineus* | Adult | Key | Canidae | *Canis lupus* | Y | 99 pools | 27 | L | Salivary Gland | *Leishmania* sp. | 27.2 | qPCR | NS |
| MA Viol | 2015 | South America | Brazil | Mato Grosso do Sul; São Paulo | Ixodidae | *Rhipicephalus sanguineus* | Adult | Key | Canidae | *Canis lupus* | Y | 99 pools | 97 | L | Gut | *Leishmania* sp. | 98.4 | immunohistochemistry "IHC" | NS |
| MA Viol | 2015 | South America | Brazil | Mato Grosso do Sul; São Paulo | Ixodidae | *Rhipicephalus sanguineus* | Adult | Key | Canidae | *Canis lupus* | Y | 99 pools | 14 | L | Ovary | *Leishmania* sp. | 13.6 | immunohistochemistry "IHC" | NS |
| MA Viol | 2015 | South America | Brazil | Mato Grosso do Sul; São Paulo | Ixodidae | *Rhipicephalus sanguineus* | Adult | Key | Canidae | *Canis lupus* | Y | 99 pools | 8 | L | Salivary Gland | *Leishmania* sp. | 7.58 | immunohistochemistry "IHC" | NS |
| Millán J, Travaini A, Zanet S, López-Bao JV, Trisciuoglio A, Ferroglio E, Rodríguez A. | 2016 | South America | Argentina | Patagonia | Ixodidae | *Amblyomma tigrinum* | Adult | Key | wild foxes | *Pseudalopex griseus* | Y | 17 monosexual pools | 11 | L | Crushed ticks | *Leishmania* sp. | 64.7 | PCR | PCR, qPCR |
| Persichetti MF, Solano-Gallego L, Serrano L, Altet L, Reale S, Masucci M, Pennisi MG. | 2016 | Europe | Italy | Southern Italy ; Sicily | Ixodidae | *Rhipicephalus sanguineus* | Adult | Key | Felidae | *Felis catus* | Y | 25 | 8 | L | Crushed ticks | *Leishmania infantum* | 10.9 | qPCR | PCR, qPCR |
| Persichetti MF, Solano-Gallego L, Serrano L, Altet L, Reale S, Masucci M, Pennisi MG. | 2016 | Europe | Italy | Southern Italy ; Sicily | Ixodidae | *Rhipicephalus pusillus* | Adult | Key | Felidae | *Felis catus* | Y | 17 |  | L | Crushed ticks | *Leishmania infantum* |  | qPCR | PCR, qPCR |
| Persichetti MF, Solano-Gallego L, Serrano L, Altet L, Reale S, Masucci M, Pennisi MG. | 2016 | Europe | Italy | Southern Italy ; Sicily | Ixodidae | *Ixodes ricinus* | Adult | Key | Felidae | *Felis catus* | Y | 10 |  | L | Crushed ticks | *Leishmania infantum* |  | qPCR | PCR, qPCR |
| Persichetti MF, Solano-Gallego L, Serrano L, Altet L, Reale S, Masucci M, Pennisi MG. | 2016 | Europe | Italy | Southern Italy ; Sicily | Ixodidae | *Ixodes ventalloi* | Adult | Key | Felidae | *Felis catus* | Y | 19 |  | L | Crushed ticks | *Leishmania infantum* |  | qPCR | PCR, qPCR |
| Persichetti MF, Solano-Gallego L, Serrano L, Altet L, Reale S, Masucci M, Pennisi MG. | 2016 | Europe | Italy | Southern Italy ; Sicily | Ixodidae | *Ixodes spp.* | Adult | Key | Felidae | *Felis catus* | Y | 2 |  | L | Crushed ticks | *Leishmania infantum* |  | qPCR | PCR, qPCR |
| Viol MA, Guerrero FD, de Oliveira BC, de Aquino MC, Loiola SH, de Melo GD, de Souza Gomes AH, Kanamura CT, Garcia MV, Andreotti R, de Lima VM, Bresciani KD. | 2016 | South America | Brazil | Mato Grosso do Sul; São Paulo | Ixodidae | *Rhipicephalus sanguineus* | Adult | Key | Canidae | *Canis lupus* | Y | 66 pools | 59 | L | Crushed ticks | *Leishmania* sp. | 89.4 | qPCR | NS |
| Viol MA, Guerrero FD, de Oliveira BC, de Aquino MC, Loiola SH, de Melo GD, de Souza Gomes AH, Kanamura CT, Garcia MV, Andreotti R, de Lima VM, Bresciani KD. | 2016 | South America | Brazil | Mato Grosso do Sul; São Paulo | Ixodidae | *Rhipicephalus sanguineus* | Adult | Key | Canidae | *Canis lupus* | Y | 66 pools | 27 | L | Ovary | *Leishmania* sp. | 40.9 | qPCR | NS |
| Viol MA, Guerrero FD, de Oliveira BC, de Aquino MC, Loiola SH, de Melo GD, de Souza Gomes AH, Kanamura CT, Garcia MV, Andreotti R, de Lima VM, Bresciani KD. | 2016 | South America | Brazil | Mato Grosso do Sul; São Paulo | Ixodidae | *Rhipicephalus sanguineus* | Adult | Key | Canidae | *Canis lupus* | Y | 66 pools | 22 | L | Salivary Gland | *Leishmania* sp. | 33.3 | qPCR | NS |
| Viol MA, Guerrero FD, de Oliveira BC, de Aquino MC, Loiola SH, de Melo GD, de Souza Gomes AH, Kanamura CT, Garcia MV, Andreotti R, de Lima VM, Bresciani KD. | 2016 | South America | Brazil | Mato Grosso do Sul; São Paulo | Ixodidae | *Rhipicephalus sanguineus* | Adult | Key | Canidae | *Canis lupus* | Y | 66 pools | 65 | L | Gut | *Leishmania* sp. | 98.5 | immunohistochemistry "IHC" | NS |
| Viol MA, Guerrero FD, de Oliveira BC, de Aquino MC, Loiola SH, de Melo GD, de Souza Gomes AH, Kanamura CT, Garcia MV, Andreotti R, de Lima VM, Bresciani KD. | 2016 | South America | Brazil | Mato Grosso do Sul; São Paulo | Ixodidae | *Rhipicephalus sanguineus* | Adult | Key | Canidae | *Canis lupus* | Y | 66 pools | 9 | L | Ovary | *Leishmania* sp. | 13.6 | immunohistochemistry "IHC" | NS |
| Viol MA, Guerrero FD, de Oliveira BC, de Aquino MC, Loiola SH, de Melo GD, de Souza Gomes AH, Kanamura CT, Garcia MV, Andreotti R, de Lima VM, Bresciani KD. | 2016 | South America | Brazil | Mato Grosso do Sul; São Paulo | Ixodidae | *Rhipicephalus sanguineus* | Adult | Key | Canidae | *Canis lupus* | Y | 66 pools | 5 | L | Salivary Gland | *Leishmania* sp. | 7.6 | Immunohistochemistry "IHC" | NS |
| MA Abdullahi, ZT Abubakar, LM Umar, ... | 2016 | Africa | Nigeria | Kaduna | Ixodidae | *Amblyomma variegatum* | NA | Key | Cattle |  | Y | 33 | 14 | T | Whole body | *Trypanosoma congolense* | 42.4 | Parasitological analysis | NS |
| MA Abdullahi, ZT Abubakar, LM Umar, ... | 2016 | Africa | Nigeria | Kaduna | Ixodidae | *Amblyomma variegatum* | NA | Key | Cattle |  | Y | 33 | 4 | T | Whole body | *Trypanosoma congolense* | 12.1 | PCR | NS |
| Rojas-Jaimes JE, Correa-Nuñez GH, Rojas N, Cáceres-Rey O. | 2017 | South America | Peru | San Lorenzo; Pueblo Botijón | Ixodidae | *Rhipicephalus (Boophilus) microplus* | Adult | Key | Tapiridae; Tayassuidae | *Tapirus terrestris; Pecari tajacu* | Y | 41 | 3 | L | Whole body | *Leishmania (V) guyanensis* | 7.3 | PCR, HRM-PCR | NS |
| Rojas-Jaimes JE, Correa-Nuñez GH, Rojas N, Cáceres-Rey O. | 2017 | South America | Peru | San Lorenzo; Pueblo Botijón | Ixodidae | *Amblyomma spp.* | Adult | Key | Tapiridae; Tayassuidae | *Tapirus terrestris; Pecari tajacu* | Y | 40 | 0 | L | Whole body | *Leishmania (V) guyanensis* | 0 | PCR, HRM-PCR | NS |
| Karasartova D, Gureser AS, Gokce T, Celebi B, Yapar D, Keskin A, Celik S, Ece Y, Erenler AK, Usluca S, Mumcuoglu KY, Taylan-Ozkan A. | 2018 | Asia | Turkey | Anatolia | Ixodidae | *Hyalomma marginatum* | NA | Key | Hominidae | *Homo sapiens* | N | 164 | 0 | L | Whole body | *Leishmania* sp. | 0 | PCR | NS |
| Karasartova D, Gureser AS, Gokce T, Celebi B, Yapar D, Keskin A, Celik S, Ece Y, Erenler AK, Usluca S, Mumcuoglu KY, Taylan-Ozkan A. | 2018 | Asia | Turkey | Anatolia | Ixodidae | *Hyalomma excavatum* | NA | Key | Hominidae | *Homo sapiens* | N | 5 | 0 | L | Whole body | *Leishmania* sp. | 0 | PCR | NS |
| Karasartova D, Gureser AS, Gokce T, Celebi B, Yapar D, Keskin A, Celik S, Ece Y, Erenler AK, Usluca S, Mumcuoglu KY, Taylan-Ozkan A. | 2018 | Asia | Turkey | Anatolia | Ixodidae | *Hyalomma aegyptium* | NA | Key | Hominidae | *Homo sapiens* | N | 1 | 0 | L | Whole body | *Leishmania* sp. | 0 | PCR | NS |
| Karasartova D, Gureser AS, Gokce T, Celebi B, Yapar D, Keskin A, Celik S, Ece Y, Erenler AK, Usluca S, Mumcuoglu KY, Taylan-Ozkan A. | 2018 | Asia | Turkey | Anatolia | Ixodidae | *Hyalomma spp.* | NA | Key | Hominidae | *Homo sapiens* | N | 46 | 0 | L | Whole body | *Leishmania* sp. | 0 | PCR | NS |
| Karasartova D, Gureser AS, Gokce T, Celebi B, Yapar D, Keskin A, Celik S, Ece Y, Erenler AK, Usluca S, Mumcuoglu KY, Taylan-Ozkan A. | 2018 | Asia | Turkey | Anatolia | Ixodidae | *Haemaphysalis parva* | NA | Key | Hominidae | *Homo sapiens* | N | 41 | 0 | L | Whole body | *Leishmania* sp. | 0 | PCR | NS |
| Karasartova D, Gureser AS, Gokce T, Celebi B, Yapar D, Keskin A, Celik S, Ece Y, Erenler AK, Usluca S, Mumcuoglu KY, Taylan-Ozkan A. | 2018 | Asia | Turkey | Anatolia | Ixodidae | *Haemaphysalis punctata* | NA | Key | Hominidae | *Homo sapiens* | N | 6 | 0 | L | Whole body | *Leishmania* sp. | 0 | PCR | NS |
| Karasartova D, Gureser AS, Gokce T, Celebi B, Yapar D, Keskin A, Celik S, Ece Y, Erenler AK, Usluca S, Mumcuoglu KY, Taylan-Ozkan A. | 2018 | Asia | Turkey | Anatolia | Ixodidae | *Haemaphysalis sulcata* | NA | Key | Hominidae | *Homo sapiens* | N | 1 | 0 | L | Whole body | *Leishmania* sp. | 0 | PCR | NS |
| Karasartova D, Gureser AS, Gokce T, Celebi B, Yapar D, Keskin A, Celik S, Ece Y, Erenler AK, Usluca S, Mumcuoglu KY, Taylan-Ozkan A. | 2018 | Asia | Turkey | Anatolia | Ixodidae | *Rhipicephalus turanicus* | NA | Key | Hominidae | *Homo sapiens* | N | 34 | 0 | L | Whole body | *Leishmania* sp. | 0 | PCR | NS |
| Karasartova D, Gureser AS, Gokce T, Celebi B, Yapar D, Keskin A, Celik S, Ece Y, Erenler AK, Usluca S, Mumcuoglu KY, Taylan-Ozkan A. | 2018 | Asia | Turkey | Anatolia | Ixodidae | *Rhipicephalu bursa* | NA | Key | Hominidae | *Homo sapiens* | N | 3 | 0 | L | Whole body | *Leishmania* sp. | 0 | PCR | NS |
| Karasartova D, Gureser AS, Gokce T, Celebi B, Yapar D, Keskin A, Celik S, Ece Y, Erenler AK, Usluca S, Mumcuoglu KY, Taylan-Ozkan A. | 2018 | Asia | Turkey | Anatolia | Ixodidae | *Dermacentor marginatus* | NA | Key | Hominidae | *Homo sapiens* | N | 17 | 0 | L | Whole body | *Leishmania* sp. | 0 | PCR | NS |
| Karasartova D, Gureser AS, Gokce T, Celebi B, Yapar D, Keskin A, Celik S, Ece Y, Erenler AK, Usluca S, Mumcuoglu KY, Taylan-Ozkan A. | 2018 | Asia | Turkey | Anatolia | Ixodidae | *Ixodes ricinus* | NA | Key | Hominidae | *Homo sapiens* | N | 4 | 0 | L | Whole body | *Leishmania* sp. | 0 | PCR | NS |
| Gondard M, Delannoy S, Pinarello V, Aprelon R, Devillers E, Galon C, Pradel J, Vayssier-Taussat M, Albina E, Moutailler S. | 2020 | Island | Guadeloupe | NS | Ixodidae | *Amblyomma variegatum* | Adult | Key | Bovidae | *Bos spp.* | Y | 132 | 0 | L | Whole body | *Leishmania martiniquensis* | 0 | High-throughput microfluidic qPCR | NS |
| Gondard M, Delannoy S, Pinarello V, Aprelon R, Devillers E, Galon C, Pradel J, Vayssier-Taussat M, Albina E, Moutailler S. | 2020 | Island | Guadeloupe | NS | Ixodidae | *Rhipicephalus (Boophilus) microplus* | Adult | Key | Bovidae | *Bos spp.* | Y | 165 | 0 | L | Whole body | *Leishmania martiniquensis* | 0 | High-throughput microfluidic qPCR | NS |
| Gondard M, Delannoy S, Pinarello V, Aprelon R, Devillers E, Galon C, Pradel J, Vayssier-Taussat M, Albina E, Moutailler S. | 2020 | Island | Martinique | NS | Ixodidae | *Rhipicephalus (Boophilus) microplus* | Adult | Key | Bovidae | *Bos spp.* | Y | 281 | 2 | L | Whole body | *Leishmania martiniquensis* | 0.7 | High-throughput microfluidic qPCR | NS |
| Luu L, Bown KJ, Palomar AM, Kazimírová M, Bell-Sakyi L. | 2020 | Europe | Slovakia | Bratislava | Ixodidae | *Ixodes ricinus* | Adult | Key | None |  | Y | 9 pools (45 ticks) | 1 | T | Whole body | *Trypanosoma caninum* | 11.1 | Culture, PCR, Sequencing | NS |
| Dyonisio GHS, Batista HR, da Silva RE, Azevedo RCFE, Costa JOJ, Manhães IBO, Tonhosolo R, Gennari SM, Minervino AHH, Marcili A. | 2021 | South America | Brazil | Pará | Ixodidae | *Amblyomma cajennense* | NA | Key | Boividae | *Bubbalus spp.* | Y | 48 | 3 | T | Whole body | *Trypanosoma vivax* | 6.25 | PCR, Sequencing | NS |
| Dyonisio GHS, Batista HR, da Silva RE, Azevedo RCFE, Costa JOJ, Manhães IBO, Tonhosolo R, Gennari SM, Minervino AHH, Marcili A. | 2021 | South America | Brazil | Pará | Ixodidae | *Rhipicephalus (Boophilus) microplus* | NA | Key | Boividae | *Bubbalus spp.* | Y | 45 | 2 | T | Whole body | *Trypanosoma vivax* | 4.5 | PCR, Sequencing | NS |
| Said Y, Lahmar S, Dhibi M, Rjeibi MR, Jdidi M, Gharbi M. | 2021 | Africa | Tunisia | Nabeul;  Jendouba; Sfax; | Ixodidae | *Hyalomma excavatum* | Adult | Key | Bovidae | *Oryx dammah; Addas nascomatus; Cervus elephatus barbarus; Gazella dorcas* | N | 284 | 0 | T | Whole body | *Trypanosoma evansi* | 0 | PCR | NS |
| Said Y, Lahmar S, Dhibi M, Rjeibi MR, Jdidi M, Gharbi M. | 2021 | Africa | Tunisia | Nabeul;  Jendouba; Sfax; | Ixodidae | *Hyalomma dromedarii* | Adult | Key | Bovidae | *Oryx dammah; Addas nascomatus; Cervus elephatus barbarus; Gazella dorcas* | N | 36 | 0 | T | Whole body | *Trypanosoma evansi* | 0 | PCR | NS |
| Said Y, Lahmar S, Dhibi M, Rjeibi MR, Jdidi M, Gharbi M. | 2021 | Africa | Tunisia | Nabeul;  Jendouba; Sfax; | Ixodidae | *Hyalomma marginatum* | Adult | Key | Bovidae | *Oryx dammah; Addas nascomatus; Cervus elephatus barbarus; Gazella dorcas* | N | 2 | 0 | T | Whole body | *Trypanosoma evansi* | 0 | PCR | NS |
| Said Y, Lahmar S, Dhibi M, Rjeibi MR, Jdidi M, Gharbi M. | 2021 | Africa | Tunisia | Nabeul;  Jendouba; Sfax; | Ixodidae | *Rhipicephalus bursa* | Adult | Key | Bovidae | *Oryx dammah; Addas nascomatus; Cervus elephatus barbarus; Gazella dorcas* | N | 2 | 0 | T | Whole body | *Trypanosoma evansi* | 0 | PCR | NS |
| Said Y, Lahmar S, Dhibi M, Rjeibi MR, Jdidi M, Gharbi M. | 2021 | Africa | Tunisia | Nabeul;  Jendouba; Sfax; | Ixodidae | *Rhipicephalus sanguineus sensu lato* | Adult | Key | Bovidae | *Oryx dammah; Addas nascomatus; Cervus elephatus barbarus; Gazella dorcas* | Y | 18 | 1 | T | Whole body | *Trypanosoma evansi* | **5.5** | PCR | NS |
| Said Y, Lahmar S, Dhibi M, Rjeibi MR, Jdidi M, Gharbi M. | 2021 | Africa | Tunisia | Nabeul;  Jendouba; Sfax; | Ixodidae | *Ixodes ricinus* | Adult | Key | Bovidae | *Oryx dammah; Addas nascomatus; Cervus elephatus barbarus; Gazella dorcas* | N | 2 | 0 | T | Whole body | *Trypanosoma evansi* | 0 | PCR | NS |
| Krishnamoorthy P, Sudhagar S, Goudar AL, Jacob SS, Suresh KP. | 2021 | Asia | India | Karnataka; Kerala | Ixodidae | *Hyalomma spp.* | Adult | Key and Molecular | Bovidae | *Bos spp.* | N | 35 pools | 0 | T | Whole body | *Trypanosoma evansi* | 0 | PCR, Sequencing | NS |
| Krishnamoorthy P, Sudhagar S, Goudar AL, Jacob SS, Suresh KP. | 2021 | Asia | India | Karnataka; Kerala | Ixodidae | *Rhipicephalus (Boophilus) spp.* | Adult | Key and Molecular | Bovidae | *Bos spp.* | Y | 157 pools | 10 | T | Whole body | *Trypanosoma evansi* | 4.2 | PCR, Sequencing | NS |
| Krishnamoorthy P, Sudhagar S, Goudar AL, Jacob SS, Suresh KP. | 2021 | Asia | India | Karnataka; Kerala | Ixodidae | *Haemaphysalis spp.* | Adult | Key and Molecular | Bovidae | *Bos spp.* | N | 13 pools | 0 | T | Whole body | *Trypanosoma evansi* |  | PCR, Sequencing | NS |
| Azarmi S, Zahraei-Ramazani A, Mohebali M, Rassi Y, Akhavan AA, Azarm A, Dehghan O, Elikaee S, Abdoli R, Mahmoudi M. | 2022 | Asia | Iran | Esfahan | Ixodidae | *Rhipicephalus sanguineus* | Adult | Key | Muridae | *Rhombomys opimus; Nesokia indica* | Y | 1 | 1 | L | Whole body | *Leishamania major* | 100 | PCR, Sequencing | NS |
| Opazo A, Bacigalupo A, Urrutia S, Chávez G. | 2022 | South America | Chile | Cuesta La Dormida | Ixodidae | *Rhipicephalus sanguineus s.l.* | Nymph and Adult | Key | Canidae | *Canis lupus familiaris* | Y | 82 | 5 | T | Whole body | *Trypanosoma cruzi* | 6.0 | PCR, Sequencing | NS |
| Opazo A, Bacigalupo A, Urrutia S, Chávez G. | 2022 | South America | Chile | Cuesta La Dormida | Ixodidae | *Amblyomma tigrinum* | Nymph and Adult | Key | Canidae | *Canis lupus familiaris* | N | 11 | 0 | T | Whole body | *Trypanosoma cruzi* | 0 | PCR, Sequencing | NS |
| Mumcuoglu KY, Arslan-Akveran G, Aydogdu S, Karasartova D, Kosar N, Gureser AS, Shacham B, Taylan-Ozkan A. | 2022 | Asia | Israel | Jerusalem | Ixodidae | *Hyalomma aegyptium* | Adult | Key | Testunidae, Camelidae | *Testudo graeca; Camelus dromedarius* | Y | 31 pools (84 ticks) | 12 pools | L | Whole body | *Leishmania infantum* | 38.7 | PCR, Sequencing | NS |
| Mumcuoglu KY, Arslan-Akveran G, Aydogdu S, Karasartova D, Kosar N, Gureser AS, Shacham B, Taylan-Ozkan A. | 2022 | Asia | Israel | Jerusalem | Ixodidae | *Hyalomma dromedarii* | Adult | Key | Testunidae, Camelidae | *Testudo graeca; Camelus dromedarius* | Y | 9 pools (75 ticks) | 5 pools | L | Whole body | *Leishmania infantum* | 55.6 | PCR, Sequencing | NS |
| Mumcuoglu KY, Arslan-Akveran G, Aydogdu S, Karasartova D, Koşar A, Savci U, Keskin A, Taylan-Ozkan A. | 2022 | Asia | Israel | (Ashkelon, Caesarea, Ga'ash, Hadera, Mikhmoret, Nahal Alexander, Nitzanim, Olga, Hadera); (Jerusalem, Hulda, Rehovot, Beit Oved); (Re'im, Revivim, Ze'elim) | Ixodidae | *Rhipicephalus sanguineus s.l.* | Adult | Key | Testunidae, Canidae, Erinaceedae, Mustelidae, No host (Flagging) | *Canis familiaris; Erinaceus concolor; Meles mele; Testudo graeca* | Y | 65 pools (374 ticks) | 6 | L | Whole body | *Leishmania infantum* | 9.2 | PCR, Sequencing | NS |
| Mumcuoglu KY, Arslan-Akveran G, Aydogdu S, Karasartova D, Koşar A, Savci U, Keskin A, Taylan-Ozkan A. | 2022 | Asia | Israel | (Ashkelon, Caesarea, Ga'ash, Hadera, Mikhmoret, Nahal Alexander, Nitzanim, Olga, Hadera); (Jerusalem, Hulda, Rehovot, Beit Oved); (Re'im, Revivim, Ze'elim) | Ixodidae | *Rhipicephalus turanicus* | Adult | Key | Testunidae, Canidae, Erinaceedae, Mustelidae, No host (Flagging) | *Canis familiaris; Erinaceus concolor; Meles mele; Testudo graeca* | Y | 43 pools (226 ticks) | 10 | L | Whole body | *Leishmania infantum* | 23.5 | PCR, Sequencing | NS |
| Ergunay K, Mutinda M, Bourke B, Justi SA, Caicedo-Quiroga L, Kamau J, Mutura S, Akunda IK, Cook E, Gakuya F, Omondi P, Murray S, Zimmerman D, Linton YM. | 2022 | Africa | Kenya | Isiolo county, Kalama, Lewa, Ol Pejeta, Samburu, Sangare gardens, Sarara, Solio ranch , Oljogi | Ixodidae | *Rhipicephalus spp.* | Adult | Key | Bovidae, Elephantidae, ,Giraffidae, Equidae, Rhinocerotidae, Felidae, Hyenidae | *Diceros bicornis, Syncerus caffer, Loxodonta africana, Giraffa camelopardalis, Equus grevyi, Alcelaphus buselaphus, Aepyceros melampus, Panthera pardus, Panthera leo, Equus quagga, Crocuta crocuta, Ceratotherium simum,Lycaon pictus; Bos indicus and Bos taurus* | Y | 43 pools (73 ticks) | 3 | T | Whole body | *Trypanosoma cruzi* | 6.9 | Metagenomics | NS |
| Ergunay K, Mutinda M, Bourke B, Justi SA, Caicedo-Quiroga L, Kamau J, Mutura S, Akunda IK, Cook E, Gakuya F, Omondi P, Murray S, Zimmerman D, Linton YM. | 2022 | Africa | Kenya | Isiolo county, Kalama, Lewa, Ol Pejeta, Samburu, Sangare gardens, Sarara, Solio ranch , Oljogi | Ixodidae | *Amblyomma spp.* | Adult | Key | Wild Equidae, Feliadae, Hyenidae | *Equus spp.Panthera leo, Crocuta crocuta* | N | 3 pools (3 ticks) | 0 | T | Whole body | *Trypanosoma cruzi* | 0 | Metagenomics | NS |

**Table S1.** Egger’s for publication bias

| slope | bias | se.bias | t | df | p-value |
| --- | --- | --- | --- | --- | --- |
| 0.0526 | 6.2469 | 1.5044 | 4.15 | 40 | 0.0002 |


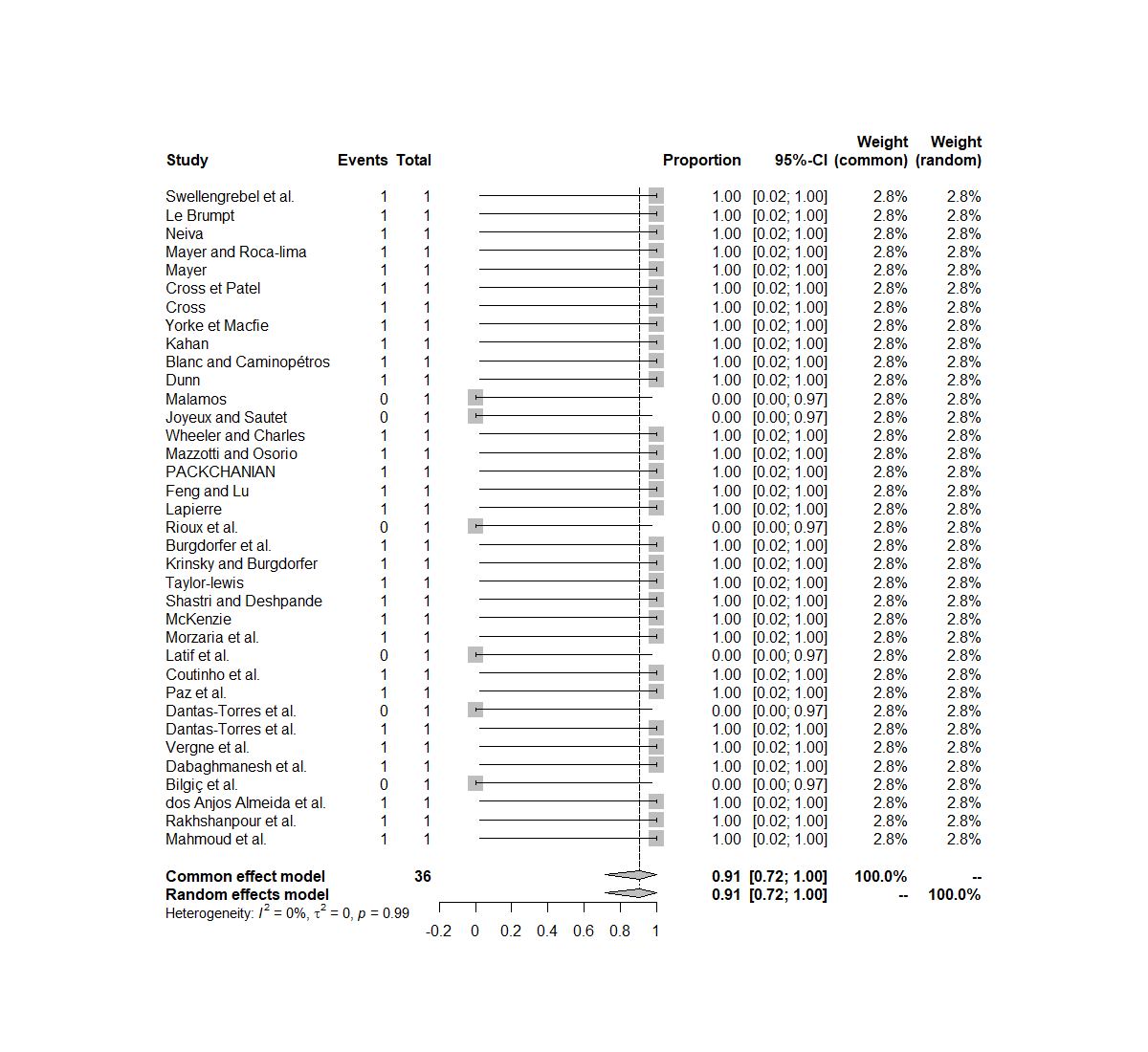


**Figure S1**: Forest plot representation of Trypanosomatidae pathogen ingestion via tick blood feeding.


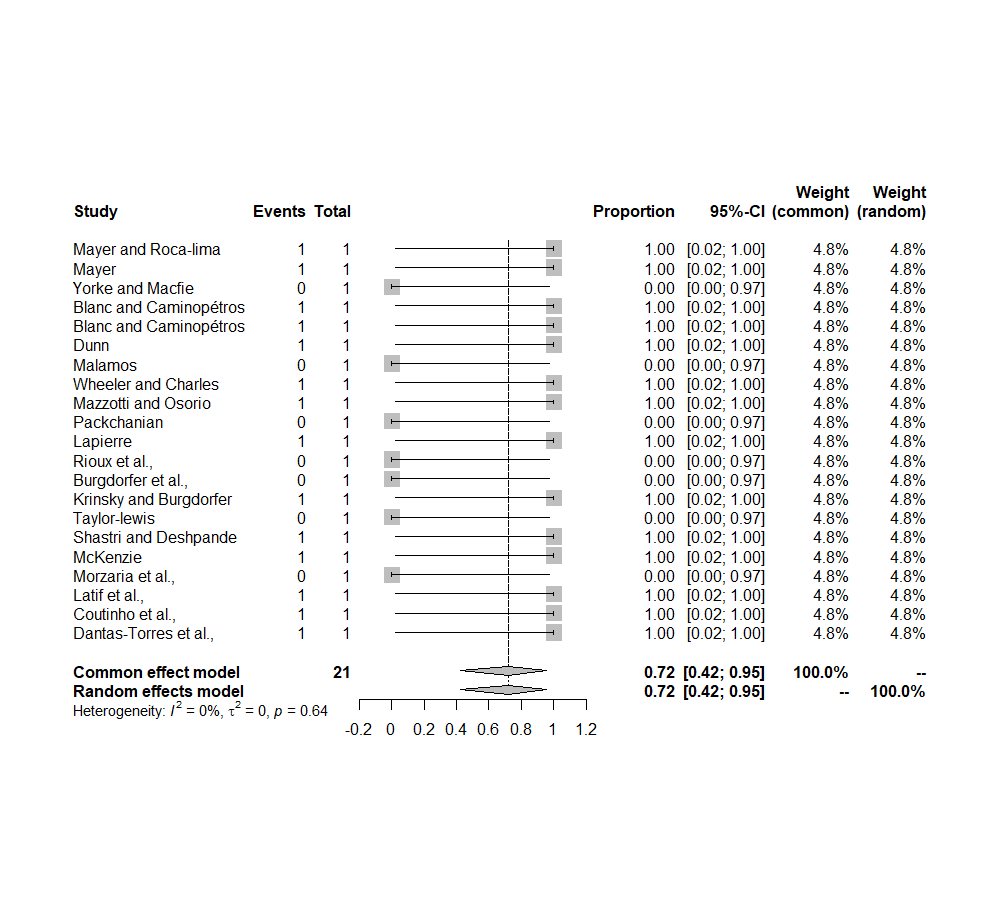


**Figure S2**: Forest plot representation of the successful attempt to transmit Trypanosomatidae via injection of tick-infected material.


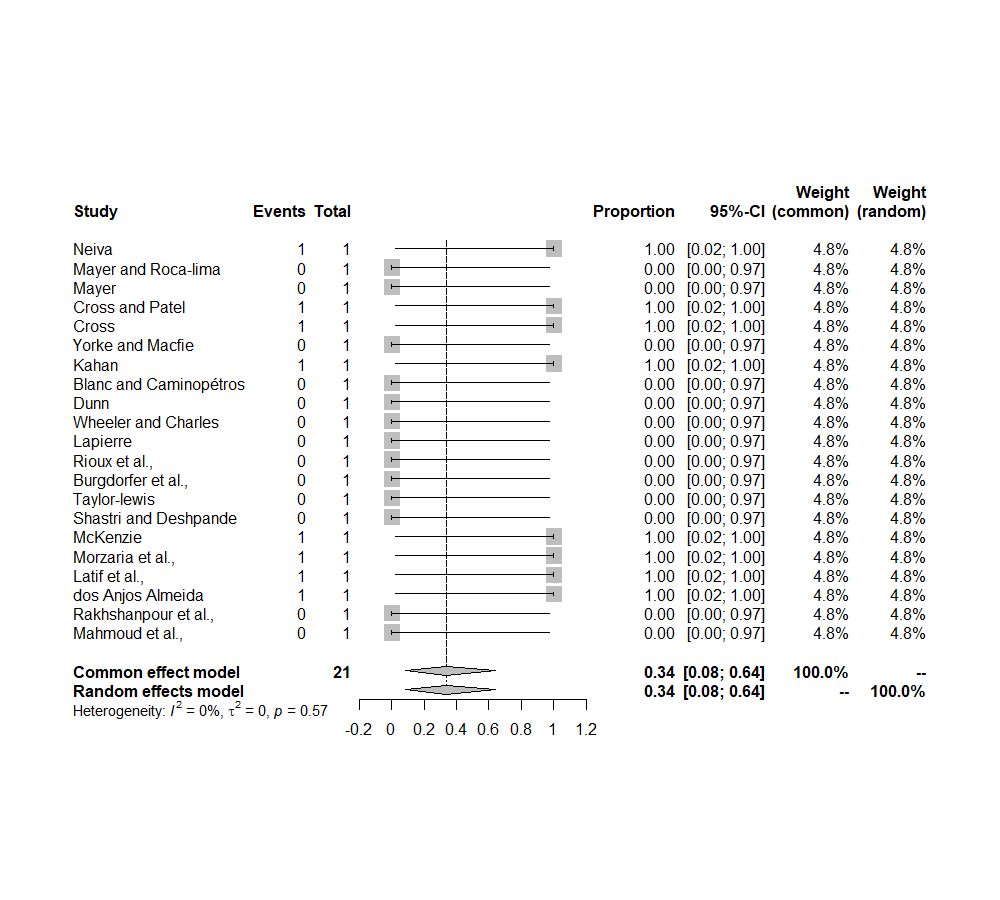


**Figure S3**: Forest plot representation of Trypanosomatidae transmission via ticks blood feeding.


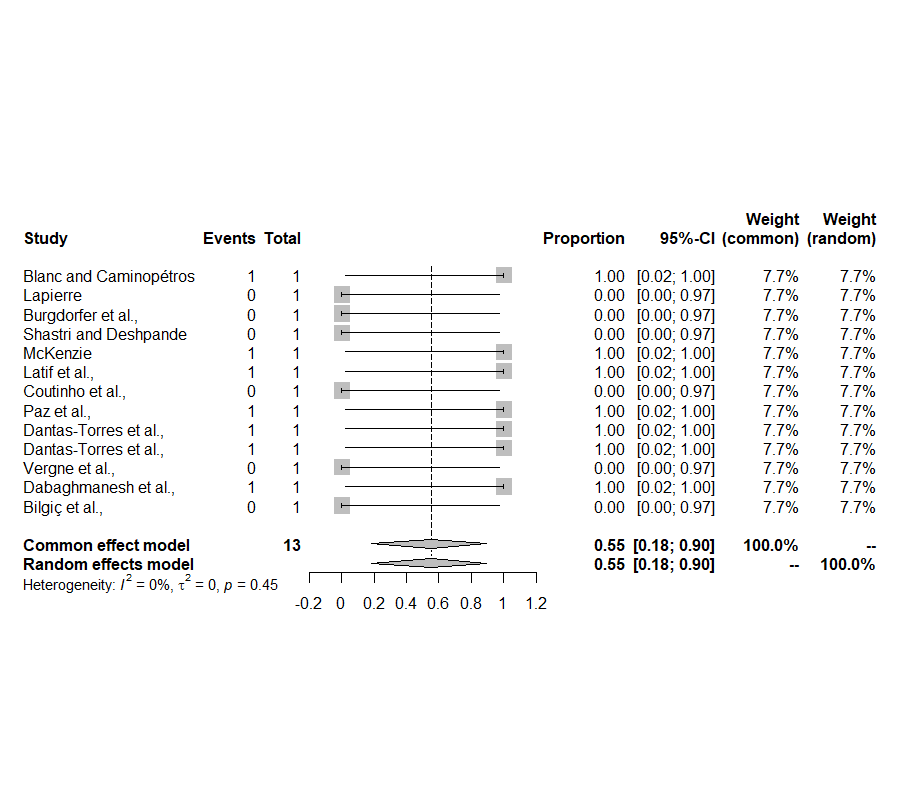


**Figure S4**: Forest plot representation of successful vertical transmission
